# Identification and Analysis of Stress-Associated Proteins (SAPs) Protein Family and Drought Tolerance of *ZmSAP8* in Transgenic *Arabidopsis*

**DOI:** 10.3390/ijms232214109

**Published:** 2022-11-15

**Authors:** Anqi Su, Qianqian Qin, Chao Liu, Jiajun Zhang, Bingxin Yu, Yifeng Cheng, Sijia Wang, Jiawen Tang, Weina Si

**Affiliations:** National Engineering Laboratory of Crop Stress Resistance Breeding, School of Life Sciences, Anhui Agricultural University, Hefei 230036, China

**Keywords:** stress-associated protein, A20/AN1 domain, maize, abiotic stress, gene family

## Abstract

Stress-associated proteins (SAPs), a class of A20/AN1 zinc finger proteins, play vital roles in plant stress response. However, investigation of SAPs in maize has been very limited. Herein, to better trace the evolutionary history of SAPs in maize and plants, 415 SAPs were identified in 33 plant species and four species of other kingdoms. Moreover, gene duplication mode exploration showed whole genome duplication contributed largely to SAP gene expansion in angiosperms. Phylogeny reconstruction was performed with all identified SAPs by the maximum likelihood (ML) method and the SAPs were divided into five clades. SAPs within the same clades showed conserved domain composition. Focusing on maize, nine *ZmSAPs* were identified. Further promoter cis-elements and stress-induced expression pattern analysis of *ZmSAPs* indicated that *ZmSAP8* was a promising candidate in response to drought stress, which was the only AN1-AN1-C2H2-C2H2 type SAP in maize and belonged to clade I. Additionally, ZmSAP8 was located in the nucleus and had no transactivation activity in yeast. Overexpressing *ZmSAP8* enhanced the tolerance to drought stress in *Arabidopsis thaliana*, with higher seed germination and longer root length. Our results should benefit the further functional characterization of *ZmSAPs*.

## 1. Introduction

Recent years, with the deterioration of the global climate and the uneven distribution of water resources, drought has become one of the major factors affecting global food production. The most effective way to ensure food security and perform plant breeding for stress tolerance is to find the key genes regulating plant stress response and cultivate new germplasms resistant to stress, such as drought stress. With the progress of research, a family of proteins called stress response proteins (SAPs) has been discovered, which are widely involved in the immune response of animals and the response to diverse biotic and abiotic stress in plants [1,2,3]. SAPs are a kind of protein harbored AN1 (ZF-AN1) zinc finger domain [Cys-X2-Cys-X(9-12)-Cys-X(1-2)-Cys-X4-Cys-X2-His-X5-His-X-Cys] and/or A20(ZF-A20) zinc finger domain [Cys-X(2^-^4)-Cys-X11-Cys-X2-Cys]. The classical SAP proteins contain an A20 (ZF-A20) at the N-terminus and an AN1 (ZF-AN1) zinc finger domain at the C-terminus, whereas some SAPs have been found to only harbor AN1 or A20 domains. Meanwhile, some SAPs also contained one or more C2H2 domains [4,5]. 

Previous studies have shown that the SAP family exists widely in different kinds of organisms, as well as in plants. According to reports, 19 SAPs have been identified in *Populus trichocarpa*, 14 in *Arabidopsis thaliana*, 18 in *Oryza sativa*, and 17 in *Medicago truncatula* [5,6,7,8,9,10,11]. Most of these genes have been reported to be involved in plant response to stress. *OsSAP1*, the first *SAP* gene identified in *O. sativa*, was shown to improve the tolerance of rice to abiotic stresses, such as cold and osmotic stress [12]. *OsSAP8* has also been reported to regulate rice drought response by interacting with rice lectin protein r40c1 (Osr40c1) [13,14]. Overexpression of *OsSAP16* could enhance drought tolerance by reducing stomatal conductance and biomass [13] while *OsSAP1/11* has been reported to improve salt resistance of rice by interacting with rice cytoplasmic kinase [15]. Similar reports have also been reported for the SAP family proteins in *A. thaliana*. *AtSAP9* has been shown to act as an ubiquitinizing enzyme that positively regulates plant resistance to osmotic stress and is also involved in ABA signaling conduction through the proteasome pathway [16]. *AtSAP10* could regulate the response of *A.thaliana* to heavy metal and high temperature stress. Overexpressing *AtSAP10* in plants could improve the tolerance of plants to heavy metal and high temperature stress and significantly increase plant biomass [17]. *AtSAP12* is involved in the regulation of plant response to salt and low temperature stress, and it has been proved to maintain the homeostasis of the REDOX state [18]. In addition, similar reports have been reported in other plants. In *Prunus persica*, *PpSAP1* regulates the response to osmotic stress, and overexpression of *PpSAP1* can improve the drought resistance of plants. In *Tamarix hispida*, *ThSAP6* is strongly induced by salt stress and has positive regulatory ability in salt stress response. In *T. hispida*, *ThSAP1* overexpressed plants showed increased salt tolerance compared with wild type plants. In *M. truncatula*, *MtSAP1* was induced to be highly expressed when embryos were exposed to drought, thereby enhancing the drought tolerance of seeds [19,20,21]. The above reports indicate that SAP proteins play important roles in plant response to abiotic stress. Therefore, understanding the evolutionary patterns, the structural and functional characteristics of the SAP family will provide us with clues for further functional characterization and in understanding the mechanism of plant stress tolerance.

Maize is one of the main food crops in the world, directly affecting global food security. Under the background of global warming, maize production has been threatened by diverse abiotic stress, such as drought and osmotic stress. Considering the crucial roles of SAPs in stress resistance, investigation of SAPs in maize is of great significance, while very few reports about SAPs in maize are available. In the present study, SAPs were genome-widely identified in maize. Moreover, their chromosomal location, evolutionary history, conserved motifs, genetic structure, and cis-acting element composition were investigated. Tissue-specific and stress-induced expression patterns of all *ZmSAPs* were conducted. Finally, one stress responsive gene, *ZmSAP8*, was selected. Its molecular feature, as well as its biological function, were studied, which will be of great help for the development of maize tolerant lines.

## 2. Results

### 2.1. Identification of Genome-Wide SAP Genes in Maize and 37 Species

**Figure 1:**
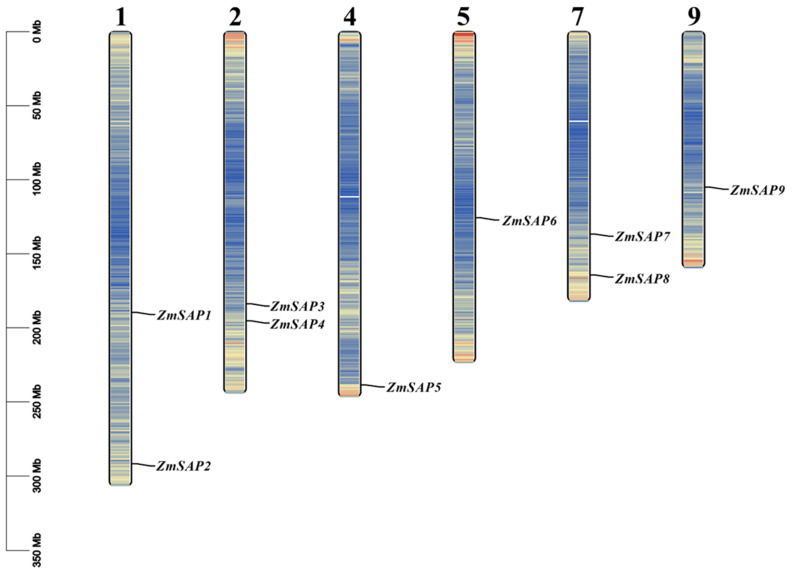
The number and location of SAP genes on maize chromosome, and the gene density on each chromosome, the number of genes from blue to red. Gene density was generated by TBtools. The parameter of bin size was set to 10^5^ bp.

**Figure 2:**
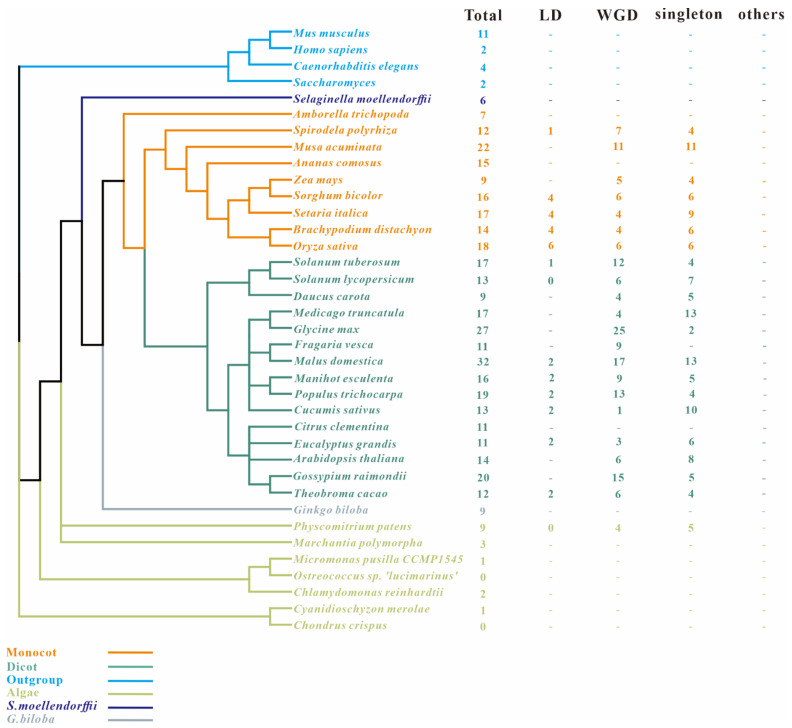
Taxonomic tree of surveyed species. Total number of genome-widely identified SAPs in each species and the numbers of SAPs involved in different duplication-modes are also listed. Species from different taxonomy and/or species are marked with different colors. “Total” represents total SAP gene numbers in each species. “LD” represents local duplication, including tandem and proximal duplication; “WGD” represents whole-genome/segmental duplication; “singleton” represents genes without paralogous pairs in MCScanX; “others” represents genes other than LD, WGD, and singleton; “-” means duplication mode was not estimated.

In the present study, nine *SAP* members were identified in maize by a genome-wide Pfam homology search (Table 1). These nine *ZmSAPs* were unevenly distributed on six out of ten chromosomes (Figure 1). There are two SAPs on chromosomes 1, 2, and 7. Chromosomes 4, 5, and 9 have one SAP, respectively. These *ZmSAPs* were further named as *ZmSAP1* to *9*, according to their order on the chromosomes. All of these *SAP* genes contain the AN1 zinc finger domain (PF01428), and five of them retain the A20 zinc finger domain (PF01754). According to the domain composition of *ZmSAPs*, *ZmSAP1*, *ZmSAP4*, *ZmSAP6*, *ZmSAP7*, *and ZmSAP9* belong to the classical A20-AN1 type SAP genes, while *ZmSAP5* and *ZmSAP2* only contained AN1. Meanwhile, *ZmSAP8* was the only AN1-AN1-C2H2-C2H2 type in maize, and *ZmSAP3* was the only AN1-AN1 type. As can be seen in Table 1, the average length of ZmSAP proteins is 179.7. The length of ZmSAP8 was biggest and has 290 amino acids. Additionally, the isoelectric points, molecular weights, and the number of AN1 and A20 domains of each SAP are also presented in Table 1. To further trace the evolutionary history of SAPs, SAP homologs were chosen from other 33 plant species ranging from unicellular algae to higher angiosperms, as well as *SAPs* from four species of other kingdoms, including *Homo sapiens (Animalia)*, *Caenorhabditis elegans (Animalia)*, *Saccharomyces cerevisiae (Chromista)*, and *Mus musculus (Animalia)*. In the analysis of the results, we found that the SAP family was evolutionary conserved and only absent in two algae, including *Chondrus crispus* and *Ostreococcus lucimarinus*. A total of 415 SAPs were identified in these surveyed species (Figure 2), with the number of SAPs in each species ranging from 1 to 32. In *Malus domestica*, most SAPs (as many as 32) were found. We also found that gene expansion existed in the SAP gene family in surveyed angiosperm species in monocot and dicot species, the SAP family numbers ranged from 9 to 32.

### 2.2. Identification of Duplication Modes of SAP Members

**Figure 3:**
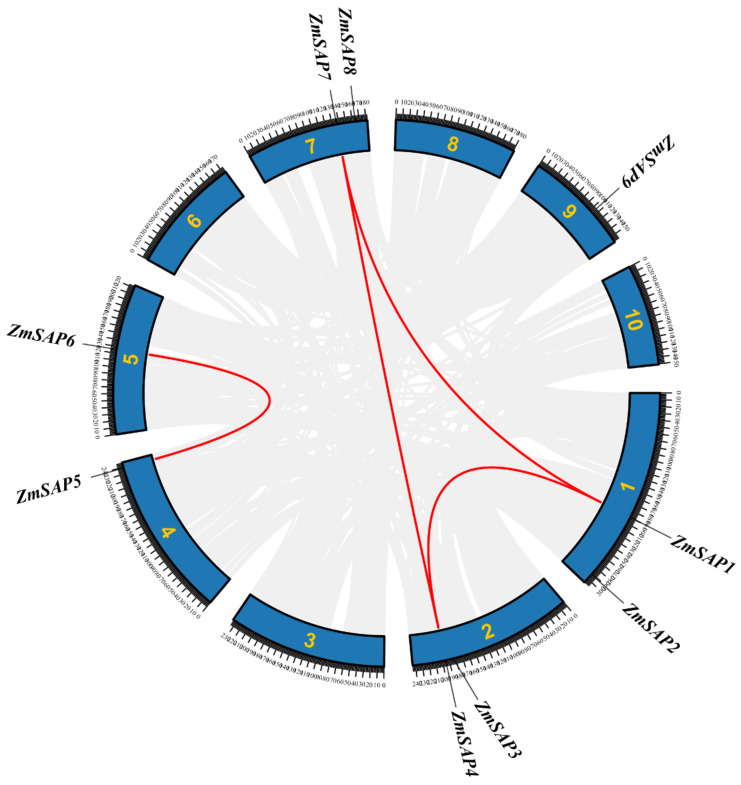
SAP collinearity analysis within the maize genome. Genome-wide collinearity genes are marked with gray lines and *ZmSAP* pairs of whole genome duplication are marked with red lines.

SAPs showed gene family expansion especially in angiosperms in the above results, which were generally shown by gene duplication events. Whole genome duplication (WGD) and local duplication (LD, including tandem and proximal duplication) are two major gene duplication modes [22]. To better trace the origins of SAPs, we investigated the gene duplication events of SAPs in angiosperms (Figure 2). The result showed that 48% of the SAPs in the angiosperms that we investigated were involved in WGD and LD events that occurred during plant evolution. We found five of the nine (55%) ZmSAPs in maize were produced by WGD events (Figure 3). Moreover, in Malus domestica, which contains the most SAPs, about 53% (17 out of 32) SAPs were produced by WGD events, and two of these were also produced by LD events (Figure 2). These results suggest that WGD and LD events play an important role in angiosperms evolution and gene expansion. 

### 2.3. Estimation of the Evolutionary Rate of SAPs in Angiosperms

**Figure 4:**
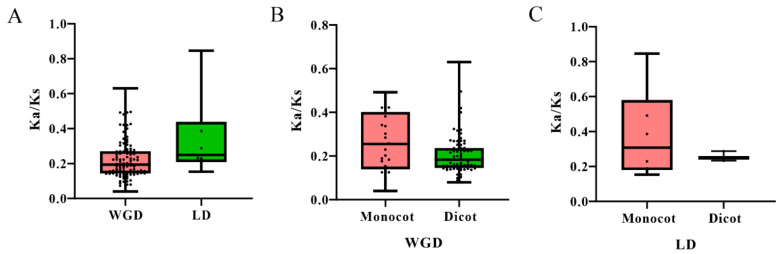
Box plot of synonymous to nonsynonymous ratios for WGD and LD duplicated pairs, (**A**) Ka/Ks values of WGD and LD SAPs gene pairs in all angiosperm plants. (**B**) Ka/Ks values of LD SAP gene pairs in dicot and monocot plants. (**C**) Ka/Ks values of WGD SAP gene pairs in dicot and monocot plants.

The ratio of non-synonymous to synonymous substitution (Ka/Ks) is one of the most important indicators in exploring the molecular evolution rate. Generally speaking, when Ka/Ks is greater than 1, positive selection is indicated. If Ka/Ks is equal to 1, neutral selection is indicated. If the value is less than 1, purifying selection is indicated. Therefore, the evolutionary rates of the SAP family were estimated by calculating the Ka/Ks of paralogous gene pairs. First, Ka/Ks values of WGD and LD SAPs gene pairs were calculated (Figure 4). Results showed that Ka/Ks values of all surveyed gene pairs were less than 1, implying purifying selection. The mean Ka/Ks ratio of WGD pairs of SAPs is smaller than that of LD pairs, indicating that the evolutionary rate of LD relatives is faster than that of WGD relatives. Moreover, the Ka/Ks ratio of WGD related gene pairs and LD related gene pairs in monocots were higher than those in dicots, indicating that the evolutionary rate of SAP genes in monocots was higher than that in dicots.

### 2.4. Phylogenetic Analysis of SAPs in All Studied Species

**Figure 5:**
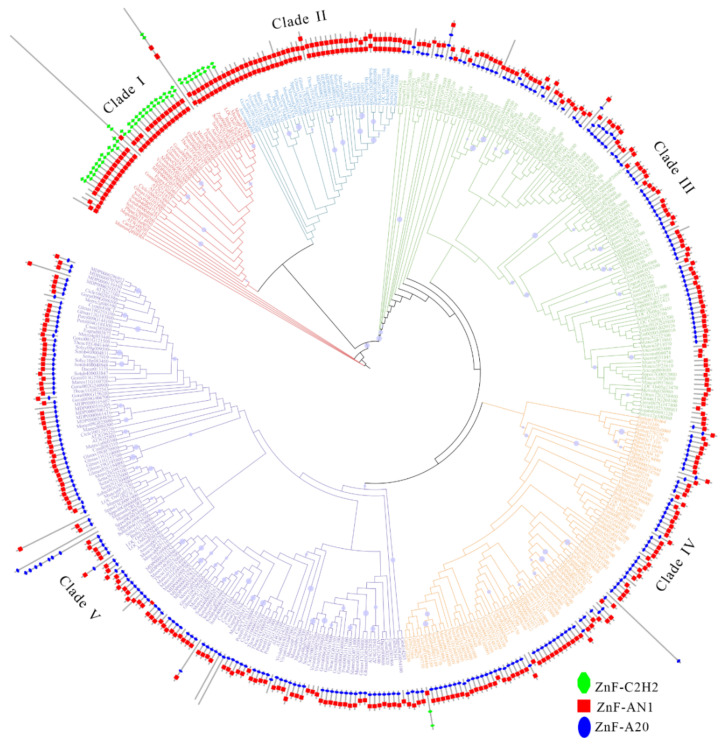
The maximum likelihood evolutionary tree was established for the selected SAPs, which contains 29 selected species and 4 outgroup species and their protein domains. Abbreviation of the species name can be found in Table S1. The Figure can be divided into five clades, and different clades are shown in different colors. Red, blue, green, orange, and purple represent clades I to V, respectively, while the topological structures on the domains are also marked, with blue ellipses representing the AN1 domain, red cuboids representing the A20 domain, and green ellipses representing the C2H2 domain.

To further investigate the evolutionary history of *SAPs* in plant kingdoms, we constructed a maximum likelihood tree with all 415 SAPs from 33 surveyed plant species (Figure 5, File S1), as well as the SAPs of four species from other kingdoms, which were regarded as outgroups. According to the topology and bootstrap values of clade nodes, the tree could be classified into five clades, named as clade I to V. Within the five clades, 39, 38, 74, 88, and 138 SAPs were clustered, respectively. Moreover, SAPs from all surveyed species presented in each clade, indicating the ancient origination of SAPs. In clade I, we found that the vast majority of SAPs were composed of the AN1AN1-C2H2-C2H2 domain, which has been reported to be closely related to plant stress response. The *AT5G48205 AT2G41835*, *AT3G57480* have been reported to be related to *A. thaliana* adversity stress resistance [18], *LOC_Os07g38240* also proved to be related to rice drought stress response [13]. No members of this clade contain the A20 domain. All SAPs in clade II were AN1-AN1 domains. *LOC_Os09g21710* [23] has been reported to be related to chloroplast development in rice. Most members of clade III contain classic A20-AN1 or A20-A20-AN1 domains, and some members of this clade have been reported to be related to plant response to stress. *AT3G12630* has been reported to improve drought and heat tolerance of plants [24]. The domain characteristics and number of members of clade IV are similar to those of clade III. As the largest clade of all clades, clade V contains the most members, and only this clade has members with multiple A20 domains. At the same time, we also found that all the members of all branches with only one or more A20 domains are from the external population, which may reflect the unique evolutionary history of the SAP family [25].

### 2.5. Gene Structure and Coding Protein Analysis of Maize SAPs

**Figure 6:**
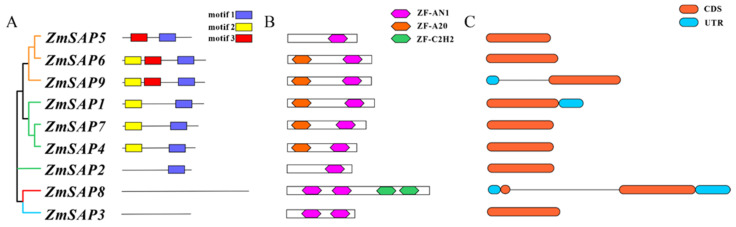
Phylogenetic and structural analysis of *ZmSAPs* members. Blue, red, green, and orange branches represent clade I to V, (**A**) motif structure of *ZmSAPs*, (**B**) protein domain of ZmSAPs, (**C**) gene structure of *ZmSAPs*.

Focusing on *SAPs* in maize, a neighbor-joining (NJ) phylogenetic tree containing only maize *SAPs* was also constructed. We found that the topology of the neighbor-joining phylogenetic tree was basically consistent with the SAP maximum likelihood phylogenetic tree (Figure 6), illustrating the accuracy of phylogeny reconstruction. By analyzing the gene structure of *SAPs* in maize, we found that most *ZmSAPs* are intron-free, only *ZmSAP1*, *ZmSAP8*, and *ZmSAP9* contain introns. Further analysis of the conserved domain and motif composition of *ZmSAPs* showed that *ZmSAPs* with close phylogenetic relationship shared similar conserved domain and motif composition. We found that *ZmSAP5*, *ZmSAP2*, *ZmSAP8*, and ZmSAP1lost the A20 domain. Moreover, *ZmSAP8* and *ZmSAP3* have two AN1 domains, and *ZmSAP8* is the only member of the family with two C2H2 domains, which have been reported to be the major regulator of plant responses to abiotic stresses such as drought [25,26].

### 2.6. Cis-Acting Element Analysis of ZmSAPs

**Figure 7:**
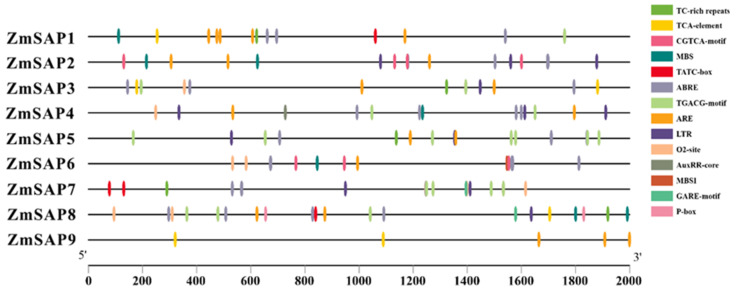
Prediction of cis-elements in the promoter regions of *ZmSAPs* genes.

The cis-acting element plays an important role in gene transcription and translation. Therefore, for further study, we used plantCARE software to perform cis-acting element analysis among the promoter regions of all *ZmSAPs* [27]. The 2000 bp upstream sequence of the *ZmSAPs* was selected for analysis (Figure 7). We found that almost all *ZmSAPs* have cis-acting elements involved in abscisic acid response (ABRE) and cis-acting elements in methyl jasmonate (MeJa) response (TGACG-motif and CGTCA-Motif). Abscisic acid (ABA) and MeJA play important roles in plant stress response and seed development [28,29]. We also detected the presence of stress response elements (TC-rich repeats) [30] in *ZmSAP2*, *ZmSAP3*, *ZmSAP6*, *ZmSAP8*, and *ZmSAP9*. On *ZmSAP1*, *ZmSAP3*, *ZmSAP4*, *ZmSAP7*, and *ZmSAP9*, we found the presence of drought-induced response elements (MBS) [31]. Low temperature stress response elements (LTRS) [32] were also found on *ZmSAP2*, *ZmSAP3*, *ZmSAP4*, *ZmSAP6*, *ZmSAP7*, *ZmSAP8*, and *ZmSAP9*. These results further supported the importance of *ZmSAPs* in plant response to abiotic stress.

### 2.7. Expression Patterns of ZmSAPs in Different Tissues

**Figure 8:**
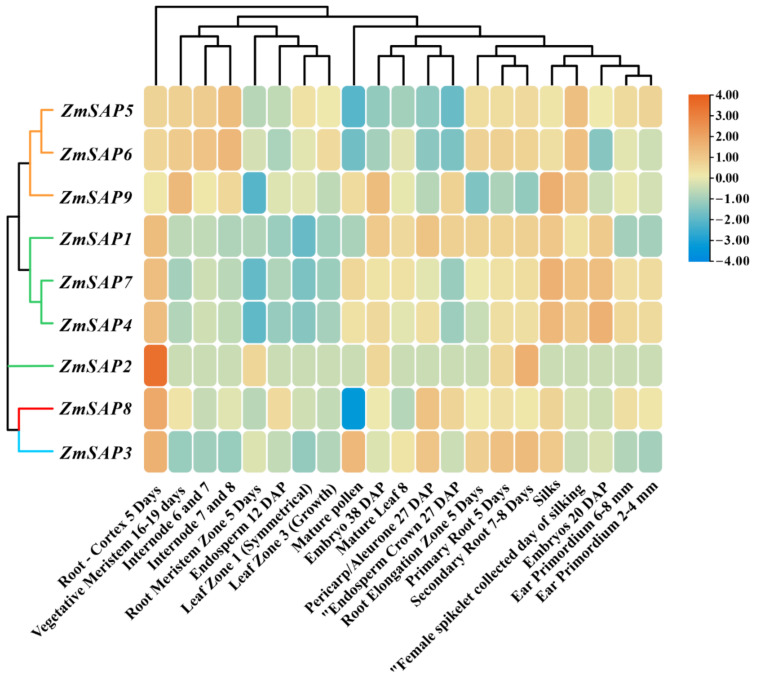
Tissue-specific expression profiles of *ZmSAP* genes. On the left, blue, red, green, and orange branch represent clade I to V, respectively. Cluster analysis of different tissues is shown at the top of Figure. The values of fragments per kilobase of exon per million fragments mapped (FPKM) were used to represent the expression levels of *ZmSAPs* in the different tissues. The heat maps were visualized by TBtools software.

To gain a better understanding of the functions of the *ZmSAP* family, we investigated the tissue expression pattern characteristics of all *ZmSAPs* at different maize tissues or at different growth stages (Figure 8). As a result, we found that the tissue expression characteristics of *ZmSAPs* were distinct. As shown in the picture, we found that all *ZmSAP* family members were highly expressed in roots. In the phylogenetic tree, *ZmSAP5*, *ZmSAP6*, and *ZmSAP9* belonging to clade IV were highly expressed in the meristem and internode, while members belonging to clade III had low expression levels in the internode. Family members within the same clade have similar expression patterns. We also found that gene pairs with WGD events had almost identical expression patterns, such as *ZmSAP5* and *ZmSAP6*, *ZmSAP4* and *ZmSAP7*.

### 2.8. Effect of Drought Stress Treatment on Relative Gene Expression Levels of ZmSAPs

**Figure 9:**
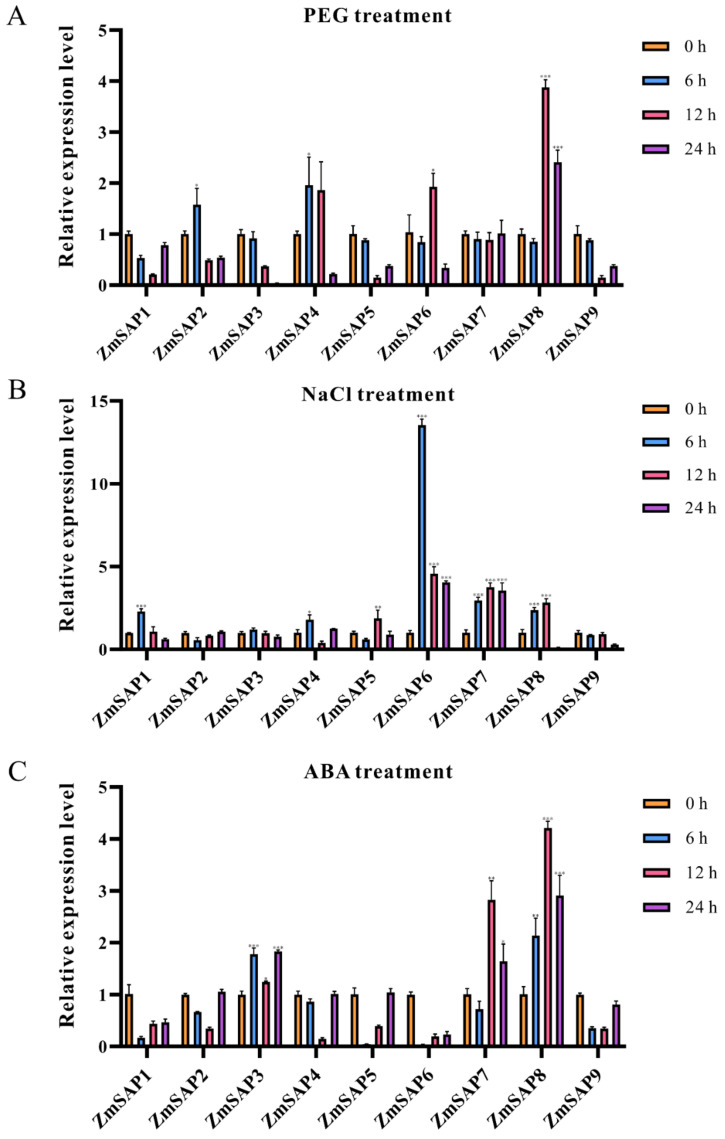
Expression patterns of nine ZmSAP genes with different treatment quantified by qRT-PCR. The relative transcript levels were determined from leaf tissues of maize subjected to (**A**) PEG: polyethylene glycol, (**B**) NaCl: salt, (**C**) ABA: abscisic acid treatment, respectively. The gene names and stress treatments are on the X-axis and relative expression levels on the Y-axis. Relative expression levels of *ZmSAP* genes were normalized to those of *ZmGADPH* and *ZmActin1* by the 2^−ΔΔCt^ method, and values at 0 h were set to 1.0. Values are presented as the mean ± standard error of values from three independent experiments. Asterisks indicate significant differences compared to the value at 0 h for each gene. (Student’s t-test; * *p* < 0.05; ** *p* < 0.01; *** *p* < 0.001).

According to our results, there are multiple abiotic stress and hormone pathway response elements in the promoter region of *ZmSAPs*, such as ABA binding element (ABRE), anaerobic response element (ARE), and drought response element (MBS) [5,15]. We examined the expression levels of *ZmSAP* family genes under treatment conditions such as polyethylene glycol (PEG), NaCl, and ABA to investigate whether their functions are related to abiotic stress [33] (Figure 9). Under PEG-simulated drought treatment conditions, only *ZmSAP8* was upregulated more than 2-fold after treatment for 12 h (Figure 9A). Under NaCl treatment, *ZmSAP6*, *ZmSAP7*, and *ZmSAP8* were up-regulated after treatment, among which the expression of *ZmSAP6* was up-regulated by more than ten-fold, and the expression of *ZmSAP7* and *ZmSAP8* was also up-regulated by more than 2-fold after 6–12 h treatments. (Figure 9B). Under ABA treatment for 6–12 h, *ZmSAP3*, *ZmSAP7*, and *ZmSAP8* were observed to be up-regulated. Moreover, only *ZmSAP7* and *ZmSAP8* were up-regulated by more than 2-fold, and *ZmSAP8* was the most up-regulated by nearly 5-fold. Based on the expression analysis results after these treatments, we concluded that the expression of *ZmSAP8* was related to a variety of abiotic stresses and abscisic acid, having great research value. Therefore, we decided to select *ZmSAP8* as a study subject to further explore its function.

### 2.9. Subcellular Localization Analysis of ZmSAP8

**Figure 10:**
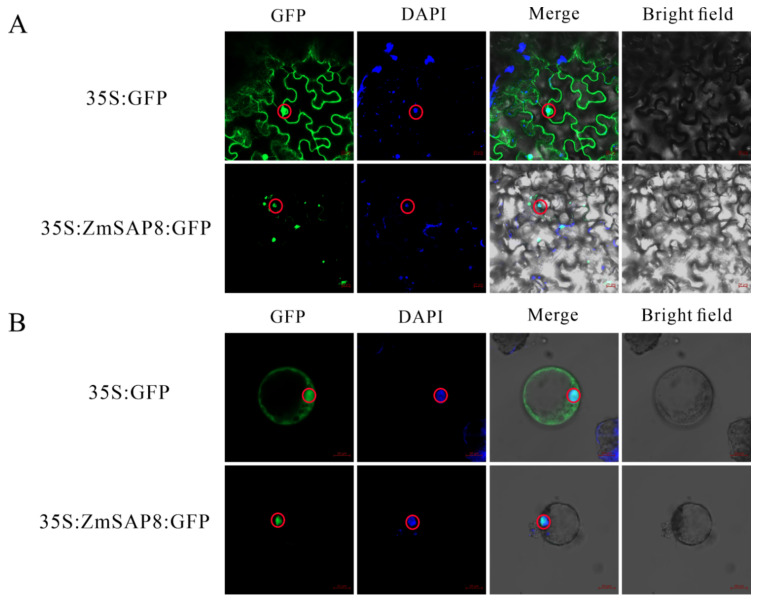
Subcellular localization of ZmSAP8. (**A**)The leaves of *Nicotiana benthamiana* were transformed with constructs harboring 35S:ZmSAP8-GFP fusion proteins via agrobacterium-mediated infiltration. The 35S::GFP empty vector was used as a positive control. Scale bar = 20 μm. (**B**) The protoplasts of maize were transformed with plasmid pCAMBIA1305 construct harboring 35S:ZmSAP8-GFP fusion proteins by PEG-mediated transformation. The 35S:GFP empty vector was used as a positive control. The nuclei in which the GFP signal and DAPI signals really overlapped are indicated with red circles. Scale bar = 10 μm. The nuclei in which the GFP signal and DAPI signals really overlapped are indicated with red circles.

Studying the localization of SAPs in cells is of great significance for the study of SAP function. We first predicted the subcellular localization of ZmSAP8 using the cell-ploc 2.0 website [34], and the predicted result was localized in the nucleus. To further confirm whether ZmSAP8 is a nuclear protein, a GFP-tagged *ZmSAP8* construct was made, which was under the control of CaMV35S promoter and fused with GFP at the C-terminus of the ZmSAP8 coding sequence (construct 35S:ZmSAP8-GFP). This construct was transiently expressed in *Nicotiana benthamiana* epidermal cells and observed by confocal laser scanning microscopy (Figure 10). The construct of 35S:GFP was applied as control and cell nuclei were stained with DAPI. As shown in Figure 10, the GFP signal of 35S:ZmSAP8-GFP construct was in the nuclei, which is overlapped with the DAPI signals, whereas the GFP signal of the control construct could be detected both in cytoplasm and nuclei. We also performed the same transformation in maize protoplasts and observed the same fluorescence signal results. The result proved that ZmSAP8 is a nuclear-localized protein.

### 2.10. Transcriptional Activity Analysis of ZmSAP8 in Yeast Cells

**Figure 11:**
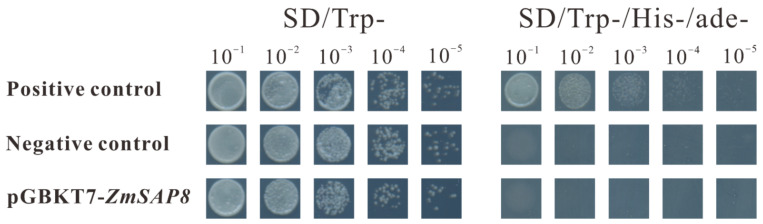
Transcriptional activity analysis of ZmSAP8, BD-p53 and Ad-SV40 large T-antigen were used as positive controls and pGBKT7 empty vector was used as negative controls.

As seen in previous reports, SAP members may function as transcription factors [35]. Thus, the yeast GAL4 system was employed to test whether ZmSAP8 has autoactivating transcriptional activity, with pGBKT7 empty vector as a negative control. BD-p53 and AD-SV40 large T antigen were used as positive control (Figure 11). All the transformed yeast cells could grow normally on SD/Trp medium, while on SD/Trp -/His-/Ade-medium, only the positive control could grow normally. However, the negative control and the yeast cells transformed with pGBKT7-ZmSAP8 recombinant vector failed to grow normally, indicating that ZmSAP8 has no transcriptional activity in yeast cells.

### 2.11. ZmSAP8 Enhances Drought Resistance in A.thaliana

**Figure 12:**
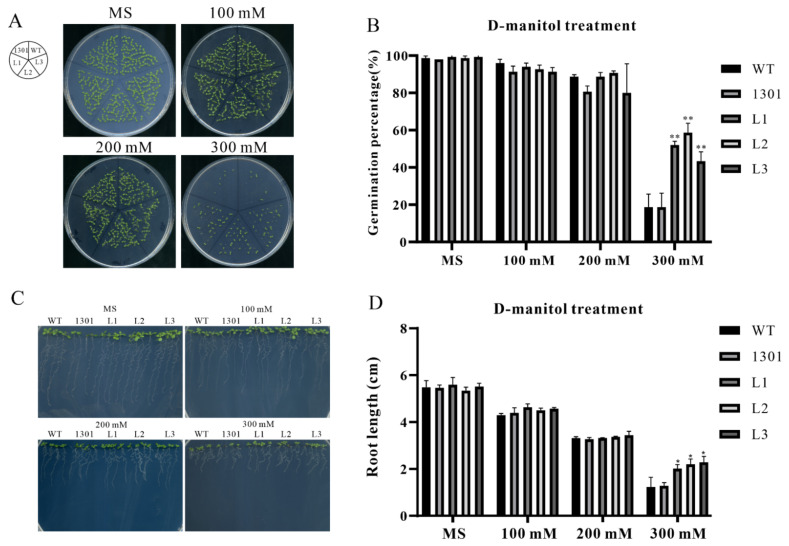
Germination assay and root length analysis of *ZmSAP8* transgenic *Arabidopsis* after mannitol treatment. Values are presented as the mean ± standard error of values from three independent experiments. (**A**) Comparison of germination percentage between transgenic (L1-L2-L3), the transgenic vector-only control (1301), and wild-type (WT) plants. (**B**) Germination statistics of transgenic (L1-L2-L3), 1301, and WT plants (**C**) Comparison of root lengths between transgenic (L1-L2-L3), 1301 and WT plants. (**D**) Root length statistics of transgenic (L1-L2-L3), 1301, and WT plants. Asterisks indicate significant differences compared to the value for WT and 1301 (Student’s *t*-test; * *p* < 0.05; ** *p* < 0.01).

To further investigate the biological function of *ZmSAP8*, it was heterologously expressed in *A.thaliana* for functional studies. Three independent homozygous T_3_ generation transgenic lines overexpressing *ZmSAP8* were screened. In our previous results, *ZmSAP8* was strongly induced by PEG-simulated drought, implying that *ZmSAP8* might be involved in plant response to drought or osmotic stress. We conducted experiments on the germination rate of transgenic *Arabidopsis* under D-mannitol simulated drought stress conditions. The results are shown in Figure 12. When the concentration of D-mannitol reaches 300 mM/L, the germination percentage of transgenic *Arabidopsis* plants (L1-L2-L3) was significantly higher than that of wild-type plants, indicating that transgenic *Arabidopsis* plants had better drought resistance than wild type plants. Because root growth under drought stress also reflects the resistance of plants to drought stress, we next determined the root length of transgenic *Arabidopsis* under D-mannitol simulated drought conditions [35]. The results are shown in Figure 12C,D. Consistent with the germination rate experiments, the root length of transgenic *Arabidopsis* was significantly longer than that of wild-type *Arabidopsis* when D-mannitol concentration reached 300 mM/L. These results indicated that ZmSAP8 enhanced resistance to drought stress in *Arabidopsis*.

## 3. Discussion

SAP proteins were first identified in humans, playing vital roles in human immune responses [36,37] and plant responses to abiotic stress. SAPs extensively exist in almost all eukaryotes. To date, they have been found in plants ranging from lower herbs to higher woody plants, including *A. thaliana*, *O. sativa*, *P. trichocarpa*, *M. domestica*, and *M. truncatula*, whereas no systematic study about the SAPs of maize has been published [6,7,16,38,39]. The present study identified SAPs from 37 species, emphasizing the evolutionary patterns of *ZmSAPs*. Results found that in these SAPs, evolutionary pattern is conserved and could be identified in nearly all the surveyed eukaryotes, including different kinds of representative plant species, *mice*, *homo sapiens*, and *Caenorhabditis elegans*. This suggests the ancient origin of SAPs. Additionally, gene expansion of SAPs was observed in angiosperms. In the long evolution, gene family expansion has been continuously occurring, generally resulting in duplication events, including WGD and LD. The duplicate events occurred and resulted in different consequences, such as dispersive repeat for genomic changes, bringing neofunctionalization or functional redundancy [40,41]. Moreover, gene family amplification also prompted gene functional change, thus adapting to the threats in the environment [42,43]. At the same time, because plants have no mobility, gene families in plants, especially those in response to stress, have more replication events [44,45]. According to the results of our gene duplication mode identification, the expansion of *SAPs* in maize is mainly due to WGD events. Five out of nine *ZmSAPs* were produced by WGD events. Moreover, there are four WGD *ZmSAP* gene pairs, whereas the *ZmSAP8* gene for our function study is not produced by the WGD event, which may be due to its distant genetic relationship with other members. The collinearity results also show that *ZmSAP8* has no collinearity with other SAP family members in maize (Figure 3).

Further phylogeny reconstruction of 415 SAPs from 37 surveyed species showed that all SAPs could be divided into five clades (Figure 5). In clade I, there were mainly SAPs from angiosperms which showed specific protein domain composition, indicating the recent origins of SAP members in clade I. Moreover, other four branches were relatively close on the evolutionary tree and only contained the AN1 domain. Thus, we hypothesized that the AN1 domain appeared first in the evolutionary history of SAPs. Meanwhile, we found that all the other members of clade I have the AN1-AN1-C2H2-C2H2 structure and do not contain the A20 domain. The C2H2 zinc finger domain has been shown to play an important role in plant response to abiotic stress [25]. *LOC_Os07g38240* (*OsSAP16*) [14] in this clade has been reported to be involved in regulating the response of rice to drought stress. *AT3G57480* (*AtSAP12*) and *AT2G41835* (*AtSAP10*) have been reported to be involved in the response of *Arabidopsis* to cold and salt stress [18]. We speculated that members in this clade may have conserved roles in response to environmental stresses, but this does not mean that members of non-clade families are not involved in abiotic stress responses. In clade IV, *MDP0000292844* (*MtSAP15*), which encoding a protein possesses the classical A20 and AN1 zinc finger domains of the SAP family, was also reported to be involved in plant response to drought stress and could enhance drought resistance in transgenic *Arabidopsis* [7]. We found that SAPs from surveyed outgroups appeared in every clade of the evolutionary tree. However, none of the SAPs from outgroups contain AN1 domain structure, and only SAPs from animals contain the multiple A20 domain structure, indicating different evolutionary events may have occurred in SAPs in animals compared with plants.

According to previous studies, cis-acting elements play an important role in plant response to abiotic stress and the regulation of growth and development [46,47]. According to our study of cis-transcription elements of *ZmSAPs*, we found that there were a variety of cis-elements involved in stress and hormone signal response in the promoter region of *ZmSAPs* (Figure 7); for example, ABRE, MBS, TCA-Element, TGACG-motif, TC-rich repeats, LTR, p-box [28,29,30,31,48,49], and other elements. First, Tc-rich repeats and MBS [30], which have been reported to be involved in plant response to stress and stress defense, are widely present in most members. This is consistent with previous reports that SAPs are extensively involved in stress responses. ABRE [28], as an ABA signaling responsive cis-acting element, is present in the promoter region of all family members except *ZmSAP9*. At the same time, we also found that CGTCA-motif, TATC-box [28,29], and other cis-elements related to jasmonic acid, salicylic acid, and other plant stress-related hormone pathways were present in the promoter region of all *ZmSAPs.* We hypothesized that the response of *ZmSAPs* to plant stress may be accomplished by regulating the plant hormone pathway [6,8,9]. Finally, *ZmSAP8*, a A20-AN1-C2H2 type *SAP* gene, was found to be responsive to PEG and salt treatment (Figure 9). Moreover, *ZmSAP8* belonged to clade I and exhibited close phylogenetic relationship with *OsSAP16* (Figure 5), *AtSAP12* and *AtSAP10*, all of which provide tolerance to diverse abiotic stresses [13,17,18]. The molecular characteristics and expression patterns of *ZmSAP8* were analyzed, and the effects of *ZmSAP8* on drought stress tolerance in transgenic *Arabidopsis* were also enhanced (Figure 12). We believe that *ZmSAP8* is a candidate gene with great application prospects and may provide major help in our subsequent development of maize lines with excellent stress resistance traits. In summary, our multi-species phylogenetic reconstruction of SAPs and phylogenetic analysis of *ZmSAPs*, together with the molecular characterization and functional study of *ZmSAP8*, provide valuable clues for revealing the functions of SAPs in plants [37] and for the development of high-quality maize strains resistant to abiotic stress in the future [50].

## 4. Method and Materials

### 4.1. Data Source and Identification of the SAP Gene Family

In this study, a total of 37 species genome data were downloaded for research, including 33 plant genomes, 4 animal genomes, and 1 microbial genome. Genome and gene annotation files of *Amborella trichopoda*, *Ananas comosus*, *Arabidopsis thaliana*, *Brachypodium distachyon*, *Caenorhabditis elegans*, *Chlamydomonas reinhardtii*, *Citrus clementina*, *Cyanidioschyzon merolae*, *Daucus carota*, *Eucalyptus grandis*, *Ginkgo biloba*, *Glycine max*, *Gossypium raimondii*, *Zea mays*, *M. domestica*, *Manihot esculenta*, *Marchantia polymorpha*, *Medicago truncatula*, *Micromonas pusilla CCMP1545*, *Musa acuminata*, *Oryza sativa*, *Physcomitrella patens, Populus trichocarpa, Selaginella moellendorffii, Setaria italica, Solanum lycopersicum*, *Solanum tuberosum*, *Sorghum bicolor*, *Spirodela polyrhiza*, *Theobroma Cacao*, and *Vitis vinifera* were all downloaded from Phytozome (https://phytozome-next.jgi.doe.gov/ accessed on 17 January 2022) [51]. Genome annotations for *Zea Mays* were downloaded from MazieGDB. (https://maizegdb.org/ accessed on 17 May 2022) [52], Genome annotations for *G. biloba* were downloaded from previous literature [53], and Genome annotations for *C. merolae*, *Mus musculus*, *Caenorhabditis elegans*, *Saccharomyces cerevisiae* and *Homo sapien* were downloaded from Ensembl Genome [54] (http://ensemblgenomes.org/ accessed on 17 May 2022). The local perl script “Pfam_scan pfam”, downloaded from HMMER3.1, was used to search the local pfam library (http://hmmer.org/ accessed on 30 May 2022) for the proteomes of these surveyed species [55]. The E-value was set as the default value. All candidate SAPs were selected with ZF-AN1 and ZF-A20 domains.

The Mw and pI of each of the *ZmSAPs* were estimated using the pI/Mw tool at the ExPASy website. The pI and GRAVY of the full length for *ZmSAPs* were calculated using ExPASy tools [56]. The *ZmSAPs* gene structures were displayed by comparing the coding and genomic sequences with TBtools [57].The chromosomal locations of *ZmSAPs* genes were mapped onto the maize linkage map with TBtools. The predicted subcellular localizations of *ZmSAPs* were analyzed using cell-ploc 2.0 website (http://www.csbio.sjtu.edu.cn/bioinf/Cell-PLoc-2/ accessed on 17 June 2022) [34]. The promoter sequence of *ZmSAPs* was obtained from the Phytozome database and the cis-elements were analyzed by using PlantCARE [27] (http://bioinformatics.psb.ugent.be/webtools/plantcare/html/ accessed on 27 June 2022). The phylogenetic species tree was constructed using the Taxonomy Browser online program (https://www.ncbi.nlm.nih.gov/Taxonomy/CommonTree/wwwcmt.cgi accessed on 3 June 2022).

### 4.2. Collinearity and Gene Replication Pattern Prediction

By using the MCSanX package to detect collinearity within and across species genomes [58], and by using MCSanX to explore repeat patterns of SAPs in angiosperms, MCScanX can effectively classify repeat genes in memory families based on their copy number and genome distribution. These include whole genome duplication (WGD)/local duplication (LD, including tandem and proximal duplication).

### 4.3. Calculation of the Ratio of Ka to Ks

Natural selection pressures received during evolution were predicted by calculating the synonymous to nonsynonymous ratio of SAP gene duplication pairs in angiosperm by selecting CDS sequences in genome files and translating them into amino acid sequences by Clustalw2. The aligned sequences and CDS sequences of each gene duplicated pair were submitted to PAL2NAL to estimate the Ka and Ks substitution rates with the PAML package [59].

### 4.4. Phylogenetic Analysis

SAP sequences from all surveyed species were selected and aligned using MAFFT with the auto-strategy [60]. Gaps in aligned sequences were deleted by TrimAL v1.2 using -automated 1 or -strictplus for ML and NJ trees, respectively [61]. Then, we used ProtTest3.4 to further assess the alignment sequence to select the most suitable amino acid substitution model for ML phylogenetic tree construction [62]. The best model according to AIC was JTT + G (−lnL = 111,863.18). Finally, the trimmed aligned protein sequences were submitted to phyML 4.0 to construct the ML phylogenetic tree [63]. The branch-supported measure based on fast approximate likelihood (Shimodaira–Hasegawa Approximate Likelihood Ratio Test, SH-aLRT) was used for branching. Other parameters were set according to the results of the ProtTest test (gamma shape = 1.254, amino acid frequency = observed value). The obtained tree was edited using MEGA-X and iTOL [64,65].

### 4.5. Conserved Motif Analysis

To detect conserved motifs in SAPs, the online MEME program (https://meme-suite.org/ accessed on 28 July 2022) was utilized with the command line as follows: meme all_protein_sequence. fas-o result-protein-evt 0.05-maxsize 10,000.0-nmotifs 40 [66]. The MEME program identified conserved motifs of the *ZmSAPs* with the default parameters, except that the number of motifs was 40.

### 4.6. Expression Analysis of ZmSAPs Genes in Different Tissues

The expression profiles for *ZmSAPs* genes were obtained from the MaizeGDB website (https://www.maizegdb.org/ accessed on 27 July 2022) [52], and a heat map was generated by TBtools software (https://github.com/CJ-Chen/TBtools accessed on 28 July 2022).

### 4.7. Plant Material Growth and Stress Treatment

Two-week-old seedlings of the maize (*Zea mays L.* Inbred line B73) plants were used to examine *ZmSAPs* gene expression patterns in response to PEG/NaCl/ABA stress treatments. The plants were grown in a greenhouse at 28 ± 2 ℃ and a 16 h light/8 h dark cycle at the School of Life Sciences, Anhui Agricultural University China. The treatments were 20% PEG6000 (*w*/*v*), 200 mM NaCl, and 100 mM ABA for 0 h, 6 h, 12 h, and 24 h, All treatments were formulated according to the concentration and then sprayed on the plant roots, respectively. For sampling, the third leaves of seedlings were harvested; immediately frozen in liquid nitrogen, and stored at −80 °C until further RNA extraction. Three seedlings were taken as three repeat samples. For *Arabidopsis*, the homozygous transgenic (L1-L2-L3), the transgenic vector-only control (1301) and wild-type (WT) plants were grown in a greenhouse under a 16 h day/8 h night photoperiod at 22 ℃. Each test was repeated a minimum of three times.

### 4.8. RNA Extraction and qRT-PCR Analysis

RNA was extracted by using RNAiso Plus (TaKaRa, NanJing, China, Code NO. 9108), the concentration and purity were checked with a nucleic acid concentration analyzer and agarose gel electrophoresis. The obtained RNA was reverse transcribed to complementary DNA (cDNA) using a reverse transcription kit (Vazyme, NanJing, China, R323). For RT-qPCR, each reaction had a total volume of 20 µL, consisting of 6 µL RNA-free water, 8 µL of AceQqPCR SYBR Green Master Mix (Vazyme, NanJing, China, Q111), 1 µL forward primers, 1 µL reverse primers, and 2 µL diluted cDNA. Three technical replications were performed per sample. The cycling of qPCR validation was 95 ℃ for 5 min, followed by 40 cycles of 95 ℃ for 10 s, 60 ℃ for 30 s, and 60 ℃ for 60 s. The qRT-PCR assay was conducted at least three times under identical conditions. *ZmActin1* and *ZmGAPDH* were used as internal controls, and primers were designed with oligo 7.0 (http://www.oligo.net/downloads.html accessed on 27 May 2022). The primers used for qRT-PCR are listed in Supplementary Table S3. The products lengths for qRT-PCR are listed in Supplementary Table S4. The relative expression levels of these genes were calculated by the 2^−ΔΔCt^ method and were displayed by GraphPrism [67].

### 4.9. Subcellular Localization Analysis

The coding region without terminator of *ZmSAP8* was cloned and fused to the subcellular localization vector pCAMBIA1305 (Abcam, ShangHai, China, ab275766) with green fluorescent protein (GFP) tags driven by the CaMV35S promoter. The pCAMBIA1305-*ZmSAP8* vector was obtained through the homologous recombination method by the ClonExpress MultiS One Step Cloning Kit (Vazyme, Najing, C113-01/02). Primers are listed in Table S2. For transient expression experiments, the recombinant plasmid pCAMBIA1305-ZmSAP8 was transformed into *N. benthamiana* epidermal cells by *Agrobacterium tumefaciformis* infection. The transformation was accomplished by injecting a syringe into the back of the *N. benthamiana* leaf [68]. Additionally, maize protoplasts were prepared and released from the leaf of 13-day-old maize B73 etiolated seedlings [69,70]. Then, by the PEG-mediated transformation method [70], the plasmid pCAMBIA1305 construct harboring 35S::ZmSAP8-GFP fusion proteins was transformed to maize protoplasts. While the pCAMBIA1305 vector was transformed as control, nuclei were stained using DAPI (1 µg/mL) staining solution [71]. The fluorescence signals were observed by a confocal laser scanning microscope (Zeiss LSM 800, Jena, Germany) after incubation in darkness at 22 °C for 16 h. Microscopy images were acquired with a confocal microscope with a ×20 objective and analyzed by using the ZEN 3.1 software (https://www.zeiss.com.cn/microscopy/products/microscope-software/zen.html accessed on 18 September 2022). The GFP and DAPI were detected at excitation of 488 nm and 461 nm.

### 4.10. Transcriptional Activation Assay

The corresponding vectors were transformed into yeast strains. Full-length *ZmSAP8* was cloned and ligated into the pGBKT7 vector (TaKaRa, Nanjing, China, Cat. No. 630489). Synthetic plasmids expressing each protein were transformed. The interaction of pGBKT7-53 with pGADT7-T (TaKaRa, Cat. No. 630442) was used as a positive control. Transcriptional activation was analyzed according to methods described in the literature [72]. Transformed cells were cultured on SD/-Trp and SD/-Trp/-His/-Ade plates. After 3–5 days of incubation at 30 °C, recombinant colonies were visualized.

### 4.11. Creation of ZmSAP8 Transgenic Arabidopsis

The CDS sequence of *ZmSAP8* was cloned. The product was cloned into the pMD18-T simple vector (TAKARA, Shanghai, China, Code No. 6011) and sequenced, and then subcloned into the pCAMBIA1301a (Abcam, Shanghai, China, ab275753) vector under the control of the CAMV35S promoter. The construct was introduced into *A. tumefaciens* strain LBA4404 by electroporation [73]. The shock voltage was chosen to be 1800 V and the experiments were repeated three times. Cauliflower Mosaic virus (CaMV) 35S promoter was used during *Arabidopsis* transformation. They were then transferred into *Arabidopsis* plants (Col-0) by floral immersion [74]. The T_3_ homozygous lines were chosen from independent T_2_ generations according to previous reports [75]. Briefly, T_0_ seeds with hygromycin resistance were planted by selfing to obtain independent T_1_ seeds. The T1 seeds with hygromycin resistance were grown for seeds. The seeds will be the T_2_ generation and hemizygous for the insert. After selfing, 100 seeds of at least six independent T_2_ generations were put on selection plates and the plates with a 3:1 ratio of resistant plants were selected. About 20 resistant plants of each line were grown to obtain seeds for T_3_ generation. Finally, 100 seeds for T_3_ generation were put on Murashige-Skoog (MS) (SIGMA, St Louis, LA, USA, M5519-50L) plates with hygromycin selection. If a plate was 100% resistant, this would be regarded as a homozygous plant. We obtained three homozygous transgenic lines (L1, L2, and L3). Using the same method, a homozygous T_3_ line (1301) transformed with an empty vector pCAMBIA1301a (Abcam, Shanghai, China, ab275753) was also generated as a transgenic control.

### 4.12. Statistical Analysis

Data analysis was conducted using GraphPrism software (graphpad-prism.cn). and significant differences were determined by Student’s t-test at significance levels of *p* < 0.01 (**), *p* < 0.05 (*), and *p* < 0.001 (***).

### 4.13. Germination Assay

For the germination test, plants of different transgenic lines and the WT were grown under the same conditions, and seeds were collected at the same time. Seeds were put on plates containing MS medium with gradient concentrations (0 mM/L, 100 mM/L, 200 mM/L, 300 mM/L) D-mannitol solution. Every plate contained 80 seeds of transgenic (L1-L2-L3), 1301, and WT plants, respectively. The plates were grown in a greenhouse with 22 ± 1 °C, 16 h light/8 h dark long day condition, a light intensity of 100 µmol (photons) · m^−2^· s^−1^, and 60% relative humidity at Anhui Agricultural University, China. After 7 days, the germination percentage was observed and calculated. If obvious emergence of the radicle through the seed coat, the seed was defined as germinated.

### 4.14. Root Length Assay

For the germination test, plants of different transgenic lines and the WT were grown under the same conditions, and seeds were collected at the same time. Seeds were put on plates containing MS medium with gradient concentrations (0 mM/L, 100 mM/L, 200 mM/L, 300 Mm/L) D-manitol solution. The plates were grown in a greenhouse with 22 ± 1 °C, 16 h light/8 h dark long day condition, a light intensity of 100 µmol (photons) · m^−2^· s^−1^, and 60% relative humidity at Anhui Agricultural University, China. After 10 days, the root length of each Arabidopsis line was photographed by a camera. Image J software (https://imagej.net/software/imagej accessed on 28 june 2022) was used to collect the root length data [73], and GraphPrism software (graphpad-prism.cn accessed on 14 September 2022) was used for data processing and presentation.

## 5. Conclusions

In total, 415 *SAPs* were identified from all the identified species, and nine *SAPs* were identified from the maize genome. Gene duplication mode analysis showed that WGD events were the main cause of *SAP* gene family expansion, while purification selection mainly affected *SAPs*. Based on phylogenetic analysis, domain analysis and sequence characteristics exploration, *SAPs* were divided into five clades (clades I to V). Moreover, most *ZmSAPs* were intron less and contained multiple stress-responsive cis-elements in their promoter region. The expression profile data showed that *ZmSAPs* had various expression patterns in different organs and different developmental tissues. The *ZmSAP8* was transcriptionally induced under PEG, NaCl, and ABA treatments. Meanwhile, *ZmSAP8* was the only AN1-AN1-C2H2-C2H2 type SAP in maize. The subcellular localization showed that ZmSAP8 was localized in the nucleus. Transcriptional activity analysis showed that ZmSAP8 had no transcriptional activity. The *Arabidopsis* transgenic plants overexpressing *ZmSAP8* increased plant tolerance to drought stress. Our results provide insights into the evolutionary history of SAPs in maize and other plant species, provide preliminary insights into their functions in maize, and provide a basis for studying their molecular mechanisms.

## Figures and Tables

**Table 1:** Information of *SAP* genes in maize.

Gene Name	v4 Gene ID	Chromosomal Location	Amino Acid Length	Mw/Da ^a^	pI ^b^	SAP Domain		Duplication Type ^c^
						AN1	A20	
*ZmSAP1*	*Zm00001d031423*	Chr1:189593205-189593937	176	18,749.01	9.12	1	1	WGD
*ZmSAP2*	*Zm00001d034389*	Chr1:291685815-291686300	161	16,678.85	9.53	1	0	/
*ZmSAP3*	*Zm00001d005698*	Chr2:183790605-183791147	180	19,399.08	9.08	2	0	/
*ZmSAP4*	*Zm00001d006016*	Chr2:195298613-195299098	161	16,782.85	9.19	1	1	WGD
*ZmSAP5*	*Zm00001d053671*	Chr4:238612589-238613017	142	15,272.56	8.8	1	0	WGD
*ZmSAP6*	*Zm00001d015842*	Chr5:125657037-125657552	171	18,291.03	8.28	1	1	WGD
*ZmSAP7*	*Zm00001d020926*	Chr7:136725907-136726398	163	17,195.35	9.45	1	1	WGD
*ZmSAP8*	*Zm00001d021842*	Chr7:164334705-164337536	290	32,038.18	8.58	2	0	/
*ZmSAP9*	*Zm00001d046767*	Chr9:104954887-104960193	174	18,409.12	8.48	1	1	/

^a^ Molecular weight, ^b^ Isoelectronic point, ^c^ TD represents transposed duplication, WGD represents whole-genome/segmental duplication.

## Data Availability

All data are displayed in the manuscript.

## Supplementary Materials

The following supporting information can be downloaded at: https://www.mdpi.com/article/10.3390/ijms232214109/s1.
