# Supplementary material for: Identification and Analysis of Stress-Associated Proteins (SAPs) Protein Family and Drought Tolerance of ZmSAP8 in Transgenic Arabidopsis
Source: Int J Mol Sci. 2022 Nov 15;23(22):14109. doi: 10.3390/ijms232214109 (PMC9696418; doi:10.3390/ijms232214109)
Supplement: Supplementary file 1 [file ijms-23-14109-s001.zip › File S1.pdf]

ALL SAPs Protein sequenes
>CaeleF58E10
MAEFPNLGKHCESTVCNRLDFLPIKCSGCGHFYCSEHFTFEAHNCPTGSRISVQVPICPICEK
PVPTPKDVNVDQQVNEHIQNNCQTPKRAKVYSNACTVPKCKKKELVAMNCSKCRNNYC
LSHRHERDHSCERKVGEMKINQKKSWTDSITSIARSRMNPCSAQARTEGDEALARSLQQEE
YNRVAPPHQTRNSNSNCTVS
>CaeleF22D6
MENEQQQAQTAPSCRAGCGFFGASATEGYCSQCFKNTLKRQQDTVRLTSPVVSPSSMAAT
SSALKSEPSSVDMCMKAAVSVSDETAKMDCEDIINVCDQINDDSVTVAESTAPTTITVDVPV
PVKKANRCHMCKKRVGLTGFSCRCGGLYCGDHRYDQAHNCQFDYKTMERETIRKNNPV
VVSDKVQRI
>CaeleY39A1A
MSDKHGEEDKKAKMHLKANDLLCVNGCGFYGTPQWENRCSKCWRAHQNEMKKCQDF
AKNRSLLSFDQFQERRKSTTESKSRGIKNLFKTSPIPEGGTSSPTSTSPASTPTRRRELSPDSLEA
RQQFTDFLVANLSTGMAQEIARSVKNAVNKISEMRMSSDDMSELVMSYYQYLGERIGGHSL
FDSPDCKVKVEDVMDQVEKYISTFCYSIFFCANHEEEVADMSLQDRIRSLHWVTAGFLETK
MVFKKQTVRDKIDEAISELIEINAKRSAFEKLDCLTKSCKAIFEALKESEASTSADEFLPTLIYV
LFRGNPPLIQSNVKFISRFAIPARLMSGEAAYFFTNLSCALEFARNMNHESLQMEKSEFEAYT
SGHLAPPLSVINSACNQAIYVLEGTIETITNVAKKAGSLAKNLTNMHGKSDDDLEKMLAVI
KETVDFYPTDEYMNMKQSIFAEEKETADILVSLSRQSSESGRGTLTDTQPSTPQPDTSLESPLI
AEIQAINLTSSGNQEHVEEA
>Phpat3c7_310
MATERVSQETTSQAPEGPVMCKNLCGFFGSQATMGLCSKCYRETVMQAKMTALAEQATQ
AAQATSATAAAVQPPAPVHETKLTCEVERTMIVPHQSSSYQQDLVTPAAAAPQAVKSSIAA
PSRPEPNRCGSCRKRVGLTGFKCRCGNLYCALHRYSDKHTCTYDYKAAGQEAIAKANPLV
VAEKVVKF*
>Phpat3c15_4950
MGTPALPDLGQHCSRVDCHQLDFLPFTCDACHKVFCLEHRQYKSHNCPNTKDHDVTVIV
CPVCHKSIRTVANEDENVTWDRHVRTNCDPSNYEKATKKPRCPVRGCKEILVFSNKVLCN
DCKREVCLKHRFGLDHGCEDFRKANRSNWGISDYGNMFMKSFKERKAPVVQSNGNSKLP
ANGASSVQSGSIKGAFSGLFSSVEAGINKLGLATSSSGTSHGNAVGRQTPSEPLKGSSGQASK
TEECPQCRARFANVAQLIKHVETMHDTPNQEMLDECPKCGRKFRDPIQLVNHVERDHGG
SSS*
>Phpat3s504_10
MSQGKDDTECQPPEGPLMCTNSCGFFGSATTLGMCSKCYRDYDSTEAKESSATGAEVVATS
SAPRLLVEHSLERTKSDGSYLAAHLPGDQGTSTEVGPSASGQHPCRPQAYRCFLCKKRVGL
TGFKCRCGNIFCSLHRCSDKHSCSFDYKTAGRDAIAKANPVVKADKFDKI*
>Phpat3c5_22610
MAQYGRKQDTEETNCQPPEGPVRCTNNCGFFGSAVTMGMCSKCYRDFVLTQAKTSSAKI
AETTPIVSVPKEPVADRALFQSSHFLSAQPEAAGASSGASASGQDPSRPKANRCFSCKKRVG
LTGFECRCGNLFCSAHRYSDKHSCTFDYKTAGRDAISKANPVVKADKMNKI*
>Phpat3c11_25720
MATERVTQETTSQTPEGPVMCKNVCGFFGSQATMGLCSKCYRETVMQAKMTAVAEQATQ
AAQVLPSAASSAQPPVLMEEDKSSFEADSMLIQPPQSSSHHPVEVAPVTVAPQVVVAPVATP

SRPAPNRCGSCRKRVGLTGFQCRCGHLFCALHRYSDKHSCTYDYKAAGQEAIAKANPLVV
AEKVVKF*
>Phpat3c8_25400
MEFPDLGLHCSEETCHQLDFLPFKCDGCRKDFCLEHRAYKAHSCANANHKDVSVQICPV
CAMSVKTVFGETVGLTMKKHQQSKTCDPRNYVKVTKKPKCPVRGCRELLTFSNKYCCNSC
QKTVCLRHRFPSDHACGIAPTRATAQIAAGSKFLASFASRHSDMHCGAESNRMASLYISEK
RIDQEKTPTVKP*
>Phpat3c8_230
MEFPDLGLHCSEETCHQLDFLPFKCDGCRKVFCLEHRTYKAHSCANANHKDVSVQICPVC
AMSVKTVFGETVELTMKKHQQSKTCDPRNYVKMTKKPKCPVRGCRELLTFSNKYCCNAC
QKTVCLRHRFPSDHACGIAPTRATAQTAAGSKFLASFASRHSDMDCGAESNRMASLYIFEK
KIDQEKTPTVKP*
>Phpat3c6_6640
MAQYGRKPDTEETNCQVPEGPVRCTNNCGFFGSAVTMGMCSKCYRDFVLSQSRTSSAKIA
GASPPVYETQEPVADRAQIQNSCLSSTQPEAAGASSGASASGQDPSRSRPNRCFSCKKRLGL
TGFECRCGNLFCSAHRYSDKHSCTFDYKLAGRDSISKANPVVKADKINKI*
>Phpat3c25_8490
MAQDNWKQEKEETNCQAPDAPIMCTNNCGFFGSAVTLGMCSKCYRDFVLTQAKSSSGKG
GEKGDTSCAAQKVAVGQRGVERTQGESVYLGGHVGGGQTEGGGTSSGGGASASGGDAC
RPQAHRCFSCKKRVGLTGFKCRCGNTFCSLHRYSDKHSCTFDYKTAGRDAIAKANPVVKA
DKVDKI*
>Vivin01018497001
MDPPLCVNGCGFFGTPATQNLCSKCYKDFLKEEEEAAKAKTKSMEKAMGSTVASTSSVDD
VVTSMTQLSLSSENTKKTISDDSSTKKKVERCETCKKKVGIIGFKCRCGSMFCAEHRLPEKH
ECSFDYKTMGREILKKQIPLIKPDKLEGRV*
>Vivin01019927001
MSFNPKLLQENRTSSFSFLDTAGGVGGGDKSEPKVPNRCMSCNKKVGLTGFRCKCGSTFC
GAHRYPEKHECTFDFKASGRDAIAKANPVVKADKLDRL*
>Vivin01019930001
MQSPYGHEIQLSLETKSAFCGLSWSCVGGGDKSEPKVPNRCMSCNKKVGLTGFRCKCGSTF
CGAHRYPEKHECTFDFKASGRDAIAKANPVVKADKLDRL*
>Vivin01025305001
MDHDETGCQAHPEGPILCINNCGFFGSPATMNMCSKCHKDMMLKQEQAKLASSFSFGSE
GSGEAKPKEGPNRCSTCKKRVGLTGFNCRCGHLFCATHRYSDKHDCPFDYRTAARDAIAK
ANPVVKAEKLDKI*
>Vivin01025308001
MCRDLKVPQISTLKSRMAQKRENEQATETELKVPETLTLCVQTCGFSASDKPRSRSPSPPDD
PDSTLENSDQGAVRRREVNRCSGCKRKLGLIGFRCRCGEMFCSKHRYSDRHECRFDYKAA
GREMIAKENPVVRPAKILKV*
>Vivin01032065001
MDHNETGCQAPPEAPILCINNCGFFGSAATMNMCSKCHKDLVLKQEQAKLAASSFESIVE
GSSNCNAKESMGPNRCSSCRKRVGLTGFNCRCGNIFCAVHRYSDKHACPFDYRTAARDAI
AKSNPVIKPEKLDKI*
>Vivin01032538001

MGEGTEAFPDLGRHCQRSDCNQLDFLPFKCDGCHQVFCLEHRTYKSHECPKPEPNSRKVV
VCETCSTSIETTGRDEKEEKAMLERHEKSGDCDPSKKKKPICPVRRCKEVLTFSNTSTCKTCQ
LNFCLKHRFPIEHACKQRLPSSTTGAKERWSDKFLAALGARIGNDCAKNGGRTVSPPGTSH
PVKAC*
>Vivin01033300001
MGTPEFPDLGKHCTVDDCKQIDFLPFTCDRCHQIFCLEHRSYIKHHCPTADRKDVTVVICP
LCAKGVRLIPDEDPNVTWETHVNTECDPSNYEKATKKKKCPVSGCREILTFSNTLKCRDCTI
DHCLKHRFGPDHKCPGPKKPEAAFPFIGLLNRSKKEDSKPSRAPTSSSKWATSFLNVASNVR
ASAEAGMTKLTSEFSQAWQTTRDGVTIDVCPKCSRGFRDPVSLVEHVERDHGGTSKA*
>Vivin01033341001
MESHDETGCQAPEGPILCINNCGFFGSAATMNMCSKCHKDLALKQEQAKLAASSIGSIVN
GSSSGNGKEPIEGPNRCTACRKRVGLTGFNCKCGNLFCAVHRYSDKHDCPFDYRTAARDAI
AKANPVVKAEKLDKI*
>Vivin01018530001
MSQRVNKKKHFKRILAMTFKSNILGINLKRVGNMEDPILCANGCGFFGTTATRNLCSKCYR
DFLKEEEESTKTKVMSMKKAMGPRVESTSSLDDEKEKNSESSANKRKPATRNLCSKCYGDY
LKEEGESAKAKAMSMEKAMGPRVESTSSLDYVVTSMAQLSLSSENTNKVINGDLL*
>Vivin01032063001
MVKGNSHTRLYEAHIPSPLPTFESLKIPFSEPQIFILQTLDMAQKTEKEETEFKVPETLALCVN
NCGFTGNPATNNMCQKCFSASASAAAAAAAGALKTNGLAGGDQPGSDEGRCLSCGASV
HGSARVQLRLQDRRSRGYRAGKSGSESCEDRESLNIIGQKKKNQTKIVKFRGRGSRSRSLHL
SEIVLSRSTLRESALERFVESFADIKMKKVSLVCSCWINLLFSFFLLFGFFVIHDPV*
>Zm00001d034389
MTQKRKSIGRGGVEDCGSPARAAACTSTTAASTSTTVEEKNTPAVFETTPPLWMTGPAETK
KPKIASSSSSSSSSSSSPDGGSNNAAVAQAQPQPPPANRCSACRKKVGLLGFRCCCGKTFCG
AHRYAEKHACGFDYKHAGRGRIAKENPIIVADKIAKI
>Zm00001d053671
MNMCSKCHKEMIMKQEQAQLAASSIDSIVNGGDNGKGPAIAATVGVAVPQVEEKTIAVQP
MHVAETSEAAAVIAKAKEGPNRCATCRKRVGLTGFNCRCGNTYCSMHRYSDKHDCQFDY
RTAARDAIAKANPVVKAEKLDKI
>Zm00001d020926
MAQRDKKEEPTELRAPEIALCANNCGFPGNPATQNLCQSCFSASRSSSSSSQPSPTSSPSASA
PAAAVPQPRPALLDAALQLAPPAAAAGQPVEASARTSANRCSSCRKRVGLTGFRCRCGDL
FCGAHRYSDRHGCRYDYRGAARDAIARENPVVRAAKIVRF
>Zm00001d031423
MAQRDKKVEEPTELRAPELTLCANSCGFPGNPATNNLCQACFLAATASSSASASVSPPPPSS
SSSSPAVLQFDEQQQQQNPRPRAPAASGPTEEPPRPARASAPAPAPASSSSVRRCQTCRKRV
GLTGFRCRCGDLFCGAHRYSDRHDCCFDYRAAGRDAIARDNPVVRAAKIVRF
>Zm00001d015842
MEHKEAGCQQPEGPILCINNCGFFGSAATMNMCSKCHKEMITKQEQAQLAASSIDSIVNG
GDGGKGPVIAASVNVAVPQVEQKTIVVQPMLVAETSEAAAVIPKAKEGPDRCAACRKRVG
LTGFSCRCGNMYCSVHRYSDKHDCQFDYRTAARDAIAKANPVVRAEKLDKI
>Zm00001d021842
MGTPEFPNLGKHCSVGDCNQIDFLPFTCDRCDHVFCLDHRSYTSHQCPNANMKDVTVLIC

PLCAKGVRLNPSEDPNITWDTHVNTDCDPSNYQKVMKKKKCPVPGCRETLTFSNTIRCKD
CTREHCLKHRFGPDHKCPGPRKVDSGFPFVSMLRRSQKAETRSNSSNNNGSSWWSSSLVN
AATNFKSSAEAGMQKLSTVTSQAFQKAKDGIAPNSSSSSGDLVEQCVHCPARFSTVGALIE
HVEKSHQINSQPSHGRVTIDVCPKCSKAFRDPVLLVEHVEKEHGGTSRV
>Zm00001d005698
MARRGTEAFPNLGAHCDKPDCNQLDFLPFDCDGCGKVFCAAHRTYGDHGCAKAADQG
RTVVVCPDCGDAIERLVPGQGEREILEAHVRSRRCDPGKKRKPRCPARRCREQLTFSNTQD
CKGCGRKVCLKHRFPADHDCAASAPGAAAAARRASGECGRGARKEGSGGWALPASIRSL
KIF
>Zm00001d046767
MEHKEAGCQAPEGPILCINNCGFFGSAATMNMCSKCHKEMITKQDQAKLAASSIDSIVNG
SDAVMEPVVAGSNTVVAVAQVELQTMNVQQPADVAGPSEGVAAISKGGKVGPNRCSACR
KRVGLTGFNCRCGNLYCALHRYSDKHDCKFDYRTAARDAIAKANPVVKADKLDKI
>Zm00001d006016
MAQRDKKEEPTELRAPEITLCANNCGFPGNPATQNLCQSCFSASRSPSSPTSSSSSLASAASQ
PRPAALVVDAAAVEALAAPAAAAVGQATEAAARASASRCSSCRKRVGLTGFRCRCGELFC
GAHRYSDRHGCSYDYRGAARDAIARENPVVRAAKVVRF
>Ancom031845
LPAEKPKSPCSEPSFPDPHPRKPEGETQSPVKSINRCLICRKKVGLTAFRCRCGDLFCGRHRY
SDAHDCSFDYKAAGREEIARNNPVIRASKIIKI*
>Ancom004280
MAQESWKKETDETECRTPEAPILCANNCGFFGSAVTNNLCSKCYRDYTMKQQAMAAPAV
PVADNILTFAAAAAAAAAATKTAASSSVSVESFAESSENKDETHVMVPKREDVEDQKNKQ
QANRCFMCRKKVGLTRFKCRCGGTFCGAHRYSETHKCSFDYKTAGREAIAKENPVIKAEKI
EKI*
>Ancom007788
MCRAIDSFFFLLFLIFGDSTDLRSPQDPILYFYKRSIDRPSFKEMAEEQRLQEGHRLCANNCG
FFGSSATLNLCSKCYGDLHPSLDQTLTLTLPSSPTPSPSFPSSSPPPPSSSSSSSSSPEAASGSGSA
GIGTEPGRAGPARPNRCAACRRRVGLTGFACRCGATFCGAHRHPERHTCGFDYKAAGRV
ALARANPLVKADKLPRI*
>Ancom008074
MLYSILLPAEKPKSPCSEPSFPDPHPWKPEGETQSPVKSINRCLICRKKVGLTAFRCRCGDLFC
GRHRYSDAHDCSFDYKAAGREEIARNNPKS*
>Ancom005923
MDQERTKREAKETEFQAPKDPILCANNCGFFGNPATDDLCSRCSKLRFLTELKKSKGRGVD
GRGVKSEPACEASREAPRSSKPENRCSFCNKKIGLLGFTCRCGDVFCSVHRYSDKHNCGFD
YRGAAQAKIAKANPLVKADKVDKI*
>Ancom000985
MGRGTEAFPDMGAHCEHEDCNQLDFLPFTCDGCQKAFCLEHRTYKSHGCPGSEHRSRTV
VVCEICSASVEREGGGEDDVAALERHVRSGACDPARKRKPRCPVPRCREALTFSNTATCRA
CHVSVCLRHRFPSDHACAPRVPVTLPARNGIQCGDRKARVRSPPSVKAY*
>Ancom000783
MAQRDKKEETEFRLPETLTLCANNCGFYGNPATNNMCLTCFKASAAAAASSFSSPSAPAA
AEVAVISSPPAPCSADDKPGSGPARISAAEGTAAGRRVSRCSACRRKVGLTGFRCRCGELFC

GEHRYSDRHACAFDYKAAAREAIARENPVVRAAKIVPF*
>Ancom005690
MEQDSKKREAEETEFQSPNVPILCANNCGFFGSPATNNLCSKCYKNHYLSKSKASIEALAIA
ATIEAKELAVQVDEAVKVDRSVEEGAPSVEASASSASKKPKNRCSFCNKKVGLMGFDCRC
GEVFCSAHRHFEKHGCPFDYRGAGKDAIAKANPVVKADKMEKI*
>Ancom017617
MYVQEYGSKCPFPNQRHVYLSYIDSVKYFTPGIKAASGEALRTFVYHEILIGYLDYCKKRGF
VSCSIWVCPSVKRDDYILYCHPTTQKMPRAEKLRDWYHKLISKATIDDVIVEHTNLHDRFFL
STGEHNTVTAANLPYCEGDYWPVEAEVLLKDDSGSTSQKKGAKAANDRMLRAYKRSSVE
RDPKDMSLMHKLGEKICPKKEEFIMIYLQYTCKHCHLPLMSAEKWKCTTCKMFMLCNQC
YTMEQQREHNDKHPSILKEKHSFRLVQQNALPDTDDGDEALRSEFFDTRTVFLNLCQNKQ
YQFDTLRRAKHSTIMLLYHLSNSIVGNAGNLGEQSVQEGAAHIDHEVVDNATKADNPML
PNKENQHKQTIEEGTTHHDNDIACNATLPDNSVLANKENPRKQTMEDALDALVHASQC
KAPHCTLLLCNKVKRLFHHGSRCSVHFNGGCDPCGKMWSLILRHAFSCVESDCCVPRCQ
DVKDRLRNQTTQSRAVRFSRRIQNHRLHQWLKGKRGGIEADRLFDFRFPPLRIRISTRALRA
EFAPMGTPEFPDLGKHCSVDDCKQIDFLPFTCDRCSQVKTAAKLYEVFCLQHRGYTKHLCP
NANQKDVTVLICPLCAKSVRLVPNEDPNITWESHVNSDCDPSNYQKATKKKRCPVPGCKE
TLTFSNTIRCRDCTKEHCLKHRFGPDHKCIGPKKPDPSFFPVNLLRRSQKAESVSNSAPIGSS
WWGSSFLNAASNAKASAEAGMLRLSNATTEAFQKARDGIAQSSSRFALMEQCVQCQARF
STVSDLIKHAERCHQNTVQLKRNGVTIDICPKCSKGFQDPVLLVEHVERDHGGTSKLKCME
TIIRERTLDPACRYSSLMGSPHLSSELAPPFQSSSRITANLAPESIHGSSISNFSTALGNDRSSFQ
RYRPNGQHRVVANFSSCSNGRVMDSIGSILGTNFAHQASDSSYLCSTPCNALLARAVQNN
GASSNILCVRDNGSRSLNDQTRMIFEQYNIYDNTHLSSGSEACVPWMRNSSLSALGSCRKS
NFMDKFNCESPCVTGLCWNGFYFGLRGIEAGHEAFTNFQNQFWRRMIYYKGNLLRIPNLV
TIITMYCKKKITLGSKRS*
>Ancom003147
MAQREKEETELQAPETLTLCVNNCGFYGNPATNNMCKTCFKASAASASASACACSSASAS
ATICSSPPPSVSVPEKPRADPKRSRPDPAAAQAARKVKRCARCTKRVGLTGFRCRCGELFCG
EHRYSDRHACVFDYKTAGREAIARENPLVRASKIIKI*
>Ancom000233
MAQESWKKETDETEYRTPEVPILCSNNCGFFGSAVTNNLCSKCYRDFMMKQQETAIPTVA
KVENMPTVESSSSSSSCAPIEPLVESSENNNETRLVISKSKEVEDHGKSRAANRCFMCRKKVG
LTGFKCRCGGTFCSAHRFYETHQCSFDYKTVGREAIAKENPVVKAEKIKKI*
>Ancom003480
MARESCNFDKDEAEILKPSSSSPPPSTSPPPPPPSPPLLSLRQKRKAPEIDLNEDFASSDCAHR
PDDEGESRLSVGFSNRCSACRKKVGLTSFRCRCGGLFCGRHRHSEAHRCSFDYKAAGREEI
ARANPLIRAAKIIKI*
>Ancom030346
MADQERRMEDGHRRCANNCGLFGSTATRNLCSRCYRDFRLKQQDHAAAADIACDNSRL
PSRSPSTTSSSCVTAPAPLVPTEEPASSATASAEQPQLASNGPSRCAACRKRVGLSAGFQCRC
GATFCGAHRYPERHACGFDYKAAGREAIARANPLVRADKLRRI*
>Ancom015643
MLNKSLIRGMEHEETGCQAPEGPILCINNCGFFGSAATMNMCSKCHKEVMLKEQQAKLA
ASSIDSIVNGGGSSSSGSSEKDPVAVAPKNADVAVASVEPKTVVAQTADPVGSSSDDAMKSK

AKEGPSRCATCRKRVGLTGFNCRCGSVFCGSHRYADMHKCPFDYQTAGRDEIAKANPVVK
AEKLDKI*
>Ancom004688
MAGERCNLDKDEAEILKPSSSSSPSSSSSSPPPSSSSSSPPLFLKPPQESPAQRPEALATATAASA
AATEAVAAAVSAAAAAPKPDELRLPAAARSISRCSTCKKRVGLTGFRCRCGELFCGRHRYS
DTHDCSFDYKALGREEIIKNNPVVRASKIIKI*
>Thcac1EG024711
MGTPEFPDLGKHCSVEDCKQIDFLPFTCDRCRLVFCLEHRSYIKHRCPKADKKDVTVVICPL
CAKGVHLIPDEDPNITWETHVNTECDPSNYDKVTKKKKCPVPGCREVLTFSNTIKCRDCTI
DHCLKHRFGPDHNCPGPKKPDSGFPFMGLLSRSRKEESKPNRAPATSSTNWATSFRNAAST
VRATAEASMTKLSSELSQKWQIARDGAGLSGSSSSSSNGSTAQVEECPQCGAKFSSVTTLVE
HVEKVHERNNQSRVFKMSIDVCPRCSKGFRDPVALVEHVERDHGGTSKA*
>Thcac1EG024819
MILMSWRLEKMESHDETGCQAPEGPILCVNNCGFFGSAATMNMCSKCHKAMILKQEQV
QLAASSIGSIVNGSSSGNGKEPTLAAALDVQSGIFESKNGSAEPSIDPSRMTFGGMKIKEGPN
RCTTCRKRVGLTGFNCRCGNLFCAAHRYSDKHDCPFDYRTAARDAIAKANPVVRAEKLD
KI*
>Thcac1EG022543
MAEEHRCQAPEGHRLCVNNCGFFGSPATMNLCSKCYRDFRLKEQQEASSIKSSLSSSPTSSS
TVVESVSQVPLLTLPEVNGESPVPAVEIAPATAEQRPQQQPNRCMVCRKRVGLTGFRCKCGI
TFCGSHRYPENHGCSFDFKTIGREEIARANPVVKAEKLEKI*
>Thcac1EG025639
MANANLPPLCAKGCGFYSSSQTKNLCSKCYNDFLKELIAKSTAEVKVDPSSAAPNPSVSVD
SSSVPTPSKLKNRCESCNKKVGLMGFSCRCGKVLCGVHRYPKEHSCNFDFKTADRLILAEE
NSLVMADKLESRI*
>Thcac1EG040596
MDEIIFACMSRRRGEEMDHEKTGCQAPPERPILCINNCGFFGSAATMNMCSKCHKDMILK
QEQAKLAASSIDNIVNGSSTGNGNETVIATGVDVPNSVEPKTILVQTSCPSGSGESIEAKLKE
GPIRCSTCKKRVGLTGFKCRCGNLFCTSHRYSDKHDCPFDYRTAARDAIAKANPVVKAEKL
DKI*
>Thcac1EG041446
MAEEYRSQAPQLCANNCGFFGIPATQNLCSKCYRDLQLKKQQSSSAKQAINQTLVPLSSPSS
SLPSSSSISSSPFSGSLPMKEVETDEEVKVEEIQIQVRPNRCLACKKRVGLTGFTCRCGMVFCG
THRHPEQHDCTFDFKEMGKEQIAKANPVVKGEKLQKI*
>Thcac1EG005908
MAQRTEKEETEFKVPETLTLCINNCGVTGNPATNNMCQKCFNATTATPSSSSSSSSSATSPSA
TGGAIAGGASIPKFSDDQSSRSTPSRSQQNRSDSSPPTTAATVTNSRTTAWNRSGNDPAAAA
EKKVVNRCSGCRKRVGLTGFRCRCGELFCAEHRYSDRHDCSYDYKTAGREAIARENPVVK
AAKIIRV*
>Thcac1EG005905
MEQNQRGCQAPKLCANNCGFFGTAATMNLCSKCHKDLVMKQQGDKLASSPNGSILDGS
PGNNHEPVSVAVDPQSTSVKSTAITAQANSASISMNTGREEEVKRGPNKCSICGKRVGLTGF
TCRCGDLFCAVHRYSDKHDCPYDYHCAARDAIAKANPVLKANKLDKI*
>Thcac1EG000958

MANIDLPPLCAKGCGFYGSSETKNLCSKCYNDFLKELVSKSKSEPKVDTALTASCPSVPVDS
SLASAPSKLKNRCESCNKKVGLMGFSCRCGKVLCDVHRYPQEHLCNFDFKKADRLILVKE
NPIIKADKLDSRI*
>Thcac1EG016105
MGSEQNEGTSFPPSEPKLCANGCGFFGTAANMNLCSKCYRDLRAGEEQAAKAKAAMEKS
LSVKTKQEDVVVETILDVKPVEELPHVGSSSTAVEQPAVVAAGNEQAEPKVSNRCFICRKKV
RLTGFKCRCGSTFCGEHRYPEKHECLFDFKGAGRDAIAKANPVVKADKVERF*
>Thcac1EG015004
MESVESMDRKLCAKACSFNGSAQRNNLCSQCYKDFLVGEFQNHNPIGEPLIPTTDQPLNSC
FTVPPYSVSHVNNSNGSVGFTFGWTNNSGGASLASTKNRCNSRNKRVGLTGFTCRCGNLF
CGKHRYPEEHECCVDLKAIGREALVKENPDCKGVGPDNLIEVYGALTIAKNQMENSVFSN
ATFLHDQNLLGDYGFSSGIMFCQIRSDMAGESHSLKGAPELPSKGVVALPSKGAVVISFGGD
LVLLDVEECQI*
>Thcac1EG016812
MVTRKRTSSNIDKNKQGDKPTQLFPLEVSQQLYITEIKHGIRFQARLGLGHKTTSLYKGLPSL
ASAQNILNSDKSLLTLSMGGGTEAFPDLGRHCQHSDCHQLDFLPFKCDGCHKVFCLEHRS
YKSHECLKSDHKSRKVVVCEICSTSIEIRAEGEEEKMVLERHEKSGDCDPTKKKKPTCPVRR
CKQILTFSNTSVCKTCRLKVCLKHRFPSEHACKQTSTAPAEAARGGWNDKFLAALASRNG
KDCAKNGRRSSSPTTPSVKAY*
>Sobic010G155901
MYPSSATATAAAGGGGGEATAAAQCSGCSAALPRAALGSAGGILMFVGTAETKDLCARCT
LEYYYRTGGLGAGGRSSTSAGRHATCPFAFAAPAAPKNKNKNAVAPPMRPKSNCRCGAC
AKKVGLLGFACRRCGGTFCSAHRHAESHGCCSFDYTPGRQERANRIDKQGIGNRV*
>Sobic010G190600
MEHKETGCQAPEGPILCINNCGFFGSAATMNMCSKCHKEMIMKQEQAKLAASSIDSIVNG
NDAVMEPVVAGNTVVAAAPIELQTMNVQPADVAGPSEGAAVISKGKVGPNRCSTCRKRV
GLTGFNCRCGNLYCALHRYSDKHDCKFDYRTAARDAIAKANPVVKADKLDKI*
>Sobic003G307500
MCAAGCGFFGSPATLGMCSVCYKKHCCTITDGPGASYAATAIDPVVTRSTATAVTPGPGAA
AAKPTVVAARTPAASVCLLAPAAKGAVAEAAAVSPPHAPEAAAKKKKAPPPGRCAACCK
KVGLTGFVCRCGNTFCGSHRYAEEHGCSFDFKGASRDAIARANPVIKAEKLTGKI*
>Sobic004G079100
MEHKEAGCQQPEGPILCINNCGFFGSAATMNMCSKCHKEMIMKQEQAQLAASSIDSIVNG
GDGGKGPVIAATVDAAVPQVEEKTIVVQPMHVAETSEAAAVIPKAKEGPNRCATCRKRVG
LTGFNCRCGNMYCSVHRYSDKHDCQFDYRTAARDAIAKANPVVKAEKLDKI*
>Sobic004G164500
MAEEQQQQQQQRWQDGHRLCANNCGFFGSPATLDLCSKCYRDLYPQEQQQPAPAGPFV
PAASAFHPSSSSVSPEPEPPAASAAGAKAGRCASCRKRVGLTGFACRCGATFCGVHRYPER
HACAFDFRAAGRDAIARANPVVKGDKLKDKI*
>Sobic007G138200
MAERQEVISGVGAAAAPMCANGCGFFGSAATNNFCSTCYKQDHLMITKTAGAGAAPVD
EKKKIEVADKAAGLVMPEQLAGGGGQDPAAAVAMSTPVARAPTGKENANGGPLETLML
QLEASGRRGLIECANACGYFGYPATNNLCTLCYRDFLESVHSSPAAADKVVVLAAEQPAA
AQISAATSSATRAVEAAAAAAASSRCASCRKKVGLLGFPCRCGGTFCALHRYAEKHACGFDF

KAAGREKIAKNNPLVVAAKINKI*
>Sobic007G138100
MAERQEVSGGMAAPMCANACGFFGSAATKNLCSKCYKEHLIKTAAVAAAPVVDEKKIEV
VAKAPPAAAHVMATATPVAQAPTTSTTEKDNGGCGCGLVETQKQEASAAVVVGGAIVKC
AADGCGFFGSSATNNMCSGCYMDFLKDAHASPAVADKVVVLAAEKQPAAAQISAATSST
APAAVKAAAAPNRCASCRKKVGLLGFPCRCGGTFCALHRYAEKHACDFDFKAAGREKIA
KNNPLVVAAKINKI*
>Sobic007G138000
MAERQQEVSGGGAAAPLCANGCGFFGSAATKNLCSKCYKEHLMIKTADDAAAAPVVDE
KKIEVVAKAPPAAAHVMPEQLLGHQDPDDATAAVAVATATPVIAQAPTSATEKENGDPVE
TENHEASGGGGGGGSIKCAANGCGFFGSTATKNMCSGCYRDFLKDAHASPAVADKVVLA
AEQLAAVQISAATSSAAPAVEAAPAAAPTNRCASCRKKVGLLGFPCRCGGTFCSLHRYAEK
HACDFDFKAAGREKIAKNNPLVVAAKINKI*
>Sobic007G212200
MAQRDKKVEEPTELRAPELTLCANSCGFPGNPATNNLCQACFQAATASSASASVSPPSPSSS
LSPSPAVFKFDEQQHARPSATAVFADRPAEQPPAPASARPIRTTSTSSSSSVNRCQSCRKRVGL
TGFRCRCGELFCGAHRYSDRHDCCFDYKAVGRDAIARENPVVRAAKIVRF*
>Sobic001G062200
MAQKRKSIDVVEDCGGHAPAARRCANGCGYFGNAATGGMCSKCYRKHAAAGATATSTS
TTTADKKTTTAQAVSETPAPAEKKAKIACAVASSSPGGGVDNAGAARAPSTEPQPVKQTAN
RCSACRKKVGLLGFRCCCGETFCGAHRYAEKHACGYDYKSAGRERIAKNNPVVVADKIAK
I*
>Sobic002G192000
MTCPDWEPSLLLAAKERKERQNERRRAMARRGTEAFPSLGAHCDEPDCNQLDFLPFDCD
GCGKVFCAAHRTYRDHGCAKAADQGRTVVVCPDCGDSIERLVPGQDEREILAAHARSRR
CDPAKNRKPRCPARRCKEQLTFSNTHHCKGCARKVCLKHRFPADHDCAASARAAAGAA
AAAARRAGGECGRGARKEGNGGGWALPASIRSLKIF*
>Sobic002G245800
MAQRDKKEEPTELRAPEITLCANNCGFPGNPATQNLCQSCFSAATASRSSSPSSPTSSSSASA
VSQPRPAALVDAAAVELLASADAAAVGQSMEVVAAAPAATAARSSAVNRCSSCRKRVGLT
GFRCRCGELFCGAHRYSDRHGCSYDYKGAGRDAIARENPVVRAAKIVRF*
>Sobic002G345300
MGTPEFPDLGKHCSVGDCNQIDFLPFTCDRCDHVFCLQHRSYTSHQCPNANMKDVTVLIC
PLCAKGVRLNPSEDPNITWDTHVNTDCDPSNYQKVTKKKKCPVPGCRETLTFSNTIRCKD
CTKEHCLKHRFGPDHKCPGPRKVDSGFPFVSMLRRSQKAETRSNSSNNNGSSWWSSSLVN
AATNFKSSAEAGMQKLSTVTSQAFQKAKDGMSPNSSSSSGDLVEQCVHCPARFSTVGALIE
HVEKSHQMNSQPSHGRVTIDVCPKCSKGFRDPVLLVEHVEREHGGTSRV*
>Sobic002G046000
MAAAGSLSRLARGHLGCGLTHKTWGVCCWLTIPLLLCSQFLVTMAHESWKQESEETGEAP
IQCINNCGFFGSSMTNKMCSKCYKDFIKLHDAPAAAAAEAVVDNKQAEEAAAQEQQQPP
NPKPPSNRCLSCRKKVGLTGFQCRCGGTFCSTHRYTDSHQCTFDYKTAAREQIAKQNPVV
MADKINKI*
>Sobic002G046100
MAQESWKQESEETGVHAPEAPILCINNCGFFGSSMTNNMCSKCYRDFIKLMETPVVEKKVI

AGASSSAVLPLEAAKRDALPTAAAAAAAEAAAAVDDKQAAQEEPPKPPSNRCLTCRKKV
GLTGFQCRCGGTFCSMHRYTDSHQCTFDYKTAAREQIAKQNPVVMAEKINKI*
>Sobic002G185000
MARRGTEAFPDLGAHCDEPDCNQLDFLPFECDGCGGFFCAAHRAYRDHGCAKAADQGR
TVVVCPDCGDAIERAAAVPGPQGDREILDAHARSRRGCDPARKRKPQCPVRRCKEALTFS
NTSQCKGCGVKVCLKHRFPADHSCAAAAAAVARRAAGTARCGRDVVQKKQGGCRPALA
VSARSFKIC*
>Solyc09g009590
MAEEHEFQSQEGGRHQLCANNCGFFGNSTTENYCSKCYRDIEKQKSDAKSIDSLFSPIKKVS
EKKIIEPIVLTTDTMKTTTSNVVTPQSNRCLVCKKKMGLMGFRCKCGTIFCGTHRYPEVHAC
TFDFKSMGREAIAKANPLIKAEKLKKI*
>Solyc01g014180
MEHNETGCQPPPEGPILCINNCGFFGSAANMNMCSKCYKDMVLKQEQAKLAVSSIENLV
NGSSASEKGMVIAGPVDVQPDTIEAQSIALPSSQTSSSSDMPDVKAKVGPNRCGTCKKKVG
ITGFKCRCGNLYCGAHRYSDKHDCLFDYRSAGQDAIAKANPVVKAEKLDKI*
>Solyc01g086970
MNMCSKCYKDMIFKQEQANFAASSIESFVNGSSNASVKAVDVAVTVQEGPAESLVIPTQVA
VPVESEQVEKAKEGPNRCSTCRRRVGLTGFNCRCGNLFCSAHRYSDKHECPYDYRKAGQD
AIAKANPVVKAEKLDKI*
>Solyc01g086960
MAQRTEKEETEFKAVPETITLCINNCGVTGNPATNNMCQKCFNATTAATSTSSSSPTGTSVTI
PHNFAEKLVRSEKSARFSSLRSSPDRKSDLDRMSQDLKKVGDTMMVKEEDQLKASLPPAKR
EVNRCSGCRRKVGLTGFRCRCGELFCGEHRYSDRHDCSYDYKTAGREAIARENPVVKAAKI
IKV*
>Solyc04g015570
MGTPEFPNLGKHCSVEDCRQIDFLPFTCDCCFKVYCLDHRSYIRHQCPTANKNNVTVVICP
LCAKGVRLNPVEDPNITWESHVNTECDPSNYEKATKKKKCPVPRCKELLTFSNTIKCRDCTI
DHCLKHRFGPDHSCAGPKKPEATFQFMNFLNGSKEDSKKAQPTTTSRWTTSLLKVVSSVKE
KFNNEFNQPQQMGQSSRATNHSVTNNSSQVEPCPQCHLRFSTVRALIDHVQKVHEKNGV
MNMTIDVCPRCSKGFRDPVALVEHVEREHKGSSM*
>Solyc12g100060
MAAQKREKEETELKVPESIPLCSPTLPVPSPSPPSTTTHLSVAVISDLKRSDRSSTESIDLKVSS
MDDQSRSTSAASPESMDLVGRKTGVKRQREANRCSGMGCRRKVGLMPFRCRCGEVFCSE
HRYSDRHDCSYDYKAAGREAIAKENPVVKAAKILKV*
>Solyc08g079700
MGSEGNKFNDGTSFPPADPILCSNGCGFFGTAATNGLCSKCYRDFKMKEDHAAMAKVAM
EKLVISRPQIESIGKVDFCSSTTSTAAERPVVEAATAEIGGSQPNRCLSCRKKVGFVGFKCRCG
STFCGTHRYPEKHDCTFDFKIKGKEEICKANPVVKADKIQRF*
>Solyc11g061770
MEHDETGCQPHPEGPILCINNCGFFGSAANMNMCSKCYKDVILKQEQEKLAASSIENFVN
GSTSQKGPVIVGSVDVQPALLESKSVVLSSPPSSSSGEAAELMAKEGPSRCSTCKKKVGLTGF
KCRCGNFYCGSHRYSDKHDCQFDYRSAARNAIAKANPVVKAEKLDKI*
>Solyc10g080200
MGTPEFPNLGKHCFVDDCRQIDFLPFTCDCCHQVFCLEHRSYNRHHCPTANNNDVTVVV

CPLCAKGVHLIPDEDPNITWESHVNTDCDPSNYEKATKKRKCPVPGCREFLTFSNTIRCREC
TVDHCLKHRFGPDHKCPGRKKPEAAFSFMNFRTGSRNGEPNKAPATSSSSWASSFFKAAEA
GMAKLGSGRGQSSNATNHSGSANRQVEQCPQCTLRFSSVTALVSHVQKVHEKNDVMNLT
VDVCPRCSKGFRDPVSLVEHVEREHKGTSKA*

>Solyc10g083460
MAEEHGFEAPEGHILCANNCGFFGSPTTQNFCSKCYNEVYIKGGQQKPIDSLFPPSQLPIPS
TSSILVLQESTAAEEEPEVVTAAVTVAVQPISAQPNRCSACRKKVGLTGFKCRCGTTFCGTHR
YPEIHGCSFDFKSIGREAIAKANPVVKAEKLGKI*

>Solyc10g079080
MESSKETGCQAPEGPILCINNCGFFGSAATMNMCSKCHKDMILKQEQAKFAATSIENIVNG
NSSSNGKEPIATGAINVQPGSADLKVISTEASSDLSSGPSSEVKPKEGPTRCTTCRKRVGLTGF
NCKCGNLFCAAHRYSDKHECPFDYKNAGRDAIAKANPVVVAEKLNKI*

>Solyc02g087210
MEGGTEAYPDLGRHCQLSDCHQLDFLPFTCHACLKVFCVEHRSCKSHECPKSDFNSRIVLV
CEICSMSMETTGCKVEDHKAILQKHEESGDCDPKKKKKKPTCPVKRCKGILTFSNTSTCKIC
RIQVCLRHRFPADHACNPTSSSSQLLLKEPNNKFLTALLARNGKDCGNKSRASSPSPANPSV
KAC*

>Solyc07g055090
MTSCGKVENPILCAKGCGFYGTSSNNNLCSQCYKAFLKEEEAKNVAVLSVKISSLTCQDDS
KGTTENIKQRCMTCKKKVGLIGFSCRCKGMFCSVHKYPEEHACTFDYKSSGRVTLATENPL
CRRDKLENRI*

>Semoe68146
GTEAFPNLGKHCSHSSCGQLDFLPFKCDACSQVFCLDHRSYTAHECPKAGAKDSTVIVCPF
CASGVKTVAGEDPNSTIERHLQTSCDPSNYDRVMKKPKCSVRGCKEVLTFSNKFHCKVCSK
NTCMKHRFPADHAC

>Semoe150464
MAQESWKREEEAAGCQLPEGPVLCANNCGFFGSVATMNLCSKCYREQSSSKAAAETMITT
TTTAAAAAATPAAKEEALAAIATSRTSTTIATAATTLVTEDDSSASQEKPRLSNRCLACRKRL
GLTGFKCRCGDVFCSMHRYSDKHNCSFDYKAAGREAIAKANPVVKADKIEKI*

>Semoe37919
AAEAPVPCANNCGFFGNGTTMNLCSKCYRDSAKMAMAVEISSGAAPILPSGSAKARASPA
AAPRDLPQSCCEGSSEGSSGSCPPPAAPDRCSCCRRKVGLMGVTCRCGKVLCFPHRYPSEH
GCEFDFQSQGRIAIAKANPVVIASKIDKI

>Semoe91519
MADIDASLDHARCANNCGYYANPGTGNLCSKCFKESIKASKAKKIEGGGGKILHPDVANV
KVGGSGAGEVEISVAEAEIGPEAKADPNRCILCSKKITLSMVFKCRCELVFCAKHRHPEDHS
CHYDYRDKGRKDISKANPVIKAEKITKI*

>Semoe270230
MAQESWKCDQDETGCQPPEGPILCANNCGFFGSKATMNLCSKCYRDVVLSQAKVSTAKN
ALEQLPLLINNTNANNNSSAATAAAATTAATIAAATTATANATGMDQGVALDEQIVLPEA
VTTSSEMNPSSGDGVADALVTSPEASGSRPPNRCNACNKRVGLTGFNCRCGNVYCALHRY
SDKHNCTYDYKSVGRDAIAKANPVVKADKIDKI*

>Semoe94262
MADIDASLDHARCANNCGYYANPGTGNLCSKCFKESIKASKAKKIEGGGKQILHPDITNV

KVSTGGDGGEEVEISVAETEIGPEAKADPNRCTSCNRKITLSMVFKCRCELVFCVKHRHPED
HSCHYDYREKGSKDISKANPVIKAEKIAKI*
>Potri009G063900
MAQRAEKEETEFKVPETLTSCINNCGVTGNPATNNMCQKCFNASTSTSNSSSSSTATSMTFA
ATATSVSNNEILKFTGEKSARSSISRSLVKDPQKSPETASDKERSCAYHVAKKEVNRCSGCRR
RVGLTGFRCRCGELFCWEHRYSDRHDCSYDYKTAGREAIARENPVVKAAKIVRV*
>Potri009G144100
MAEEQHRCQEPRLCVNNCGFFGSPATQNLCSKCYGDLRQSQPLNQLLAPSSSASVSSFSSPT
VDVIKNQIAPVLVVEGDEKGEFKAEPTVVVPQQKPNRCLTCRRRVGLTGFNCRCGMVFCG
THRYPEQHDCEFDFKSLGKEQIAKANPVVKGEKLQRI*
>Potri004G184300
MAEEQHRCQEQRLCVNNCGFYGSQATENLCSKCYRDLHQSQPLNHQLLNPSSSSAASVSS
FASPAVDVLKVNTNQKAPVVVVGDDKKDEVKAGEPAAVKQQQQPSRCLTCRRRVGLTGF
KCRCGMVFCGTHRYPEQHDCEFDFKSLGKQQIAKANPVVKGEKLQKI*
>Potri003G117100
MGSEQNDGTSFPPAEPKLCVNGCGFFGTAANMNLCSKCYRDLRAEEEQAASAKAAMEKT
LNINPKQNIDSKVVVDAPQVVVANSVQSVVSAEASSSAETVVAGGDQVPSKPANRCFSCSK
KVGLTGFQCKCGGTYCGTHRYAENHECLFDFKGAGRDAIAKANPVIKANKVERF*
>Potri003G205500
MDRDETGCQAPPERPILCINNCGFFGSAATMNMCSKCHKDMLLKQEQTKLAASSIGSIVN
GSASSNVNEPVIADTINVQINAVEPKTITVQPSCASVSGERVEAKPKEGPSRCTSCKKRVGLT
GFKCRCGDLFCASHRYSDKHDCPFDYRTAAREAIAKANPVVKAEKLDKI*
>Potri011G143600
MLVVYRFQSPRPVVVANRCNFCRREVGLTGFKCRCVYTFCSQHRYSDKHNCVFYYKSILDR
MLFLKGNSVVKQIRLIKI*
>Potri011G138500
MGGGTEAFPDLGRHCQHSECKQLDFLPFNCNGCRKVFCLEHRSYKSHECPKSDHKSRKV
VVCETCSASIETTGCNEDAEKVVLLKHEKSGDCDPRKKKKKKPTCAVKRCKEILTFSNTCTC
KTCQLKVCLKHRFPADHACKKYHPLQYM*
>Potri006G052200
MGTPQFPDLGKHCSVEDCKQIDFLPFTCDRCRQVFCLEHRSYIKHSCPKADSNGVIVVICPL
CAKGVRLNPDEDPNISWEVHVNTECDPSNYDKVTKKRKCPVRGCRELLTFSNTIKCRDCTL
DHCLKHRFGPDHTCPGPKKPDVSFPFMGLLNRSKKEESKPNRATAVSSSKWTTNFLSAAST
VRASAEAGMSKLSSEISQAWQTATNSASPSSSNGSGGMGPEECPQCGTRFSSVTNLIDHVQK
VHEKGGNQSRVLQLPMEVCPKCSKGFRDPVALVEHVERDHRDNLVEPT*
>Potri006G056500
MESHDETGCQAPEGPILCINNCGFFGSAATMNMCSKCHKDIILKQQQAQLAASSIESIVNG
NSSGNGKEPVVAVAVDVQSAPVEVKIISTEPSSATSSKPSEMKAKEGPSRCTSCRKRVGLTGF
SCRCGNLFCAVHRYSDKHNCRFDYRNAARDAIAKANPVVRAEKLDKI*
>Potri016G051700
MESHDETGCQAPEGPILCINNCGFFGSAATMNMCSKCHKDIILNQQQAQLAASSIESIVNG
NSSGNGKEPVVAGAVDVQAAPVEVKIISTEPSIASSKPSEMKAKEGPSRCTACRKRVGLTGF
GCRCGNLFCAIHRYSDKHDCPFDYRTAARDAIAKANPVVKAEKLDKI*
>Potri007G078500

MEQESQKRKLEDSSSNEAQNTPILCVNNCGFFGSPNTNNLCSKCYKEFLLTQQDTTTTTIDV
PVHVAENSAAAAAEVEGQQEGGEEEKRPVVVANRCNFCRKKVGLTGFKCRCGYTFCSQH
RYSDKHNCVFDYKSAGQDAIAKANPVVKADKIDKI*
>Potri015G131500
NQAVVSNETASTASTTASTSTVMKNRCECCNKKVGLMGFKCRCGKTFCGVHRYVKEHSCT
FDFKTYDRQNLAKQNPLVAGDKLHTRI*
>Potri015G131900
MDSQDKLTPALCAKGCGFFGSPENKNLCSKCYKDYLKEEVIAKTADKLSELVITPSSDDKNP
AVVSNETASTTTATASATTVLKNRCECCGKKVGLMGFKCRCGKTFCGVHRYAKEHSCTFDF
KTFDRQILAKQNPLVAGDKLDARI*
>Potri001G115000
MGSEQNEGTSFPSSQPQLCANGCGFFGTAANMNLCSKCYRDLRAEEEQAAFAKAAMEKT
LNMKSQQHIDSRVAVDAPQVAVANSMQSAEASSSAETAVAAGDQVSSKPANRCFSCNKKV
GLTGFMCKCGGTYCGTHRYSENHECSFDFKGAGRNAIAKANPVIKADKVGRF*
>Potri001G269400
MAQRTEKEETECKVPENLTLCINNCGVTGNPATNNMCQKCFNASTSTSNPSSSTTTTTTIT
FAATTNGVSTNEILKFTSEKSLRSSISRSPAKDHQRQPKTASDKERSDSSSVAKKEVNRCSGCR
RRVGLTGFRCRCGELFCWEHRYSDRHDCSYDYKTVGREAIARENPVVKAAKIVRV*
>Potri001G018600
MDHDETGCQAPPEGPILCTNNCGFFGSAATMNMCSKCHKGMLLKQEQANLAASSIGSIV
NGSSSSNVFEPVIADIIDVQNNAVEPKTITVQPSCASGSGERVEAKPKEGPNRCTSCKKRVGL
TGFKCRCGSLFCASHRYSDKHDCPFDYRSAAREAIAKANPVVKAEKLDKI*
>Potri012G130100
MDSINSSTLPLCAKGCGFFGSPENENFCSKCYKDYLKEGLIAEPSKKLSEPIVVTPSFDDNSP
DVVTDETTSTTTAVASTSKVKNRCECCNKKVGLMGFECRCGNTFCGVHRYPKEHSCTFDF
KTLDQQNLAKQNPLVAGDKLGSRI*
>Potri012G130000
MDSQSNLTPPLCAKDCGFFGSPEKKNLCSECYRDYVKEEESVAAETAKKLSQLVINTPSAAN
DKSPAVLTDETTSSSAAAAAAASSSTVKINRCECCNKKVGLLGFKCRCEKTFCGVHRHATE
HSCTFDFKTLGRHILAEQNPLVVSDKLHTRI*
>Potri001G269300
KGDSDAGKWSRTRPEPQGPSKAPKLCANSCGFFGTATTMNLCSKCHDDFILKQEHAKMA
LSICCVCYERFLSVDTTSNAVIAVAVDPPATSAVPLISSIPALSAAASSTDHTSIQYLSLRESFLR
AIHHYPDKHNCSSDYRSAGQDAIAKANPIVKAEKLDKI*
>LOC_Os08g39450
MAQREKKVEEPTELRAPEMTLCANSCGFPGNPATNNLCQNCFLAASASSSSSAAASPSTT
SLPVFPVVEKPRQAVQSSAAAAVALVVERPTAGPVESSSKASRSSSVNRCHSCRRRVGLTGFR
CRCGELYCGAHRYSDRHDCSFDYKSAARDAIARENPVVRAAKIVRF*
>LOC_Os08g33880
MEEQQAAAAGGGGGGGGASMCANGCGFFGSEATKKLCSKCYRDQLKAAPSSPPAAPDL
VANEEEEASTAAAAAADEQLALCSSGCGFFGSKETNNMCSKCYRDHLKATSPLFSSSSPAT
ASTTDITVPIAPATTAPTPSLKGKEEEATAAASSSAAAAAKPNRCVACRKKVGLLGFECRCG
GTFCSTHRHADKHACTFDFKKSDREKIAKENPLIVAPKITKF*
>LOC_Os07g07350

MAQESWKNESEETVHTPEAPILCVNNCGFFGSSMTNNMCSKCYRDFVKVTTMAAPVVEK
KAFTPASSSKTPLEPAKPDEVPAAAVEDKQAAQEPPKPPSNRCLSCRKKVGLTGFQCRCGG
TFCSTHRYTEAHDCTFDYKKAGRDQIAKQNPVVIAEKINKI*
>LOC_Os07g38240
MGTPEFPNLGKHCSVGDCNQIDFLPFTCDRCDHVFCLQHRSYTSHQCPNANQKDVTVLIC
PLCAKGVRLNPNEDPNITWDTHVNSDCDPSNYQKVTKKKKCPVPGCRETLTFSNTIRCKD
CTKEHCLKHRFGPDHKCPGPRKPESTFPFGNMLRRSQKAESCSNSNSSSTSSSWWSSSLLTA
ATSFKSSAEAGMQKLSTATTQAIQKAKDGISTSSSNSGDLVEQCVQCPARFSTVGALIEHCEK
SHQSNSQSSRSRVTVDVCPKCSKAFRDPVLLVEHVERDHGGTSRA*
>LOC_Os01g56040
MGQQVQHESRINVGEATHVSKAEMGANTMFATSRLNSNNKVGPELAYLSGVASSASDSST
AAPSPCYLCHKPAALHVFGLAGRYVFGSVKREAYLSQEGPRSGRTPNRIAESLPVRVVNDF
GLRLRVVTNQGPIKPRPPRPIDAIVFASIETRNRLRGFDRSFCCSAPPETYVFLPRARETIVLRA
NIIKMSSEQQASAGQPVLCASGCGFYGNPATLDMCSVCYRQHCLLNGATMATGPSSSVAA
ASAATVATGAVTSDSCSVPSAEVNGAAFSSKNNPEPATVVEKKAPANRCASCKKKVGLLGF
ACRCGATYCGTHRYPEKHACGFDFKGASRDAIARANPLIKGEKLTNKI*
>LOC_Os01g52030
MEQGSERQDERPPLPCANGCGFFGSADTRGLCSKCYRQTVMSQASAPSAAAQSAEHDQV
VLPAPEGVPVDEGAMPPPPPRHGAKTKSRCAACGRSVGLMGFECRCGAVFCGAHRYSDR
HDCGYDYRGAGRDAIARANPVVRPDKVEKL*
>LOC_Os03g57900
MASMKRKCPDDETACGSGAGAAMCVTGCGFFGSEATNNMCSRCYREHSADNDAVEEAA
AANSDLELVGVAETTTKKARMSAVVPVAVASSSSAAAEQPAAKAATAPNRCAACRKKVGL
TGFKCRCGGNFCGGHRHADAHGCGFDYKSAGKEQIAKQNPLVVADKLATRI*
>LOC_Os03g57920
MATKRKCPANGDDGGVADLEPVAGGSFASPPPEKKAKLTVAVAVAVAPSSSSATTAAAGE
ATAKREHGGFFAFARPENNTRLSVAVASSSSSASAAAEKAMAKLTVAGVAPSSSASAAAAG
KATAKREYGGFCAFARPDDKTRWRVAVASSAAAAADASYSSSSPATGEQPEANRCATCRRK
VGLTGFKCRCGGTFCGGHRYADEHGCGFDYKSSGRELIAKQNPVVVADKLAFRI*
>LOC_Os03g57890
MAQESWKKEAEETGVHTPEAPILCVNNCGFFGSRMTENMCSKCYRDTVKAKTVATVVEK
KPLASLSSTPLVTEVTDGGSGSVADGKQVMEEDTPKPPSNRCLSCRKKVGLTGFKCRCGGTF
CSMHRYADSHKCTFDYKQVGREQIAKQNPLVKADKITKI*
>LOC_Os02g32840
MAEEQRWQEGCHRLCANNCGFFGSPATLDLCSKCYRDRQGRESTAPVVVAAAASACPAT
HPSSPSSSSCPAFLPSSTAAEAGVVVAAVAKASRCASCRKRVGLTGFACRCGGTFCGAHRYP
ERHACGFDFKAAGRDAIARANPLIKGDKLKDKI*
>LOC_Os02g10200
MEHKEAGCQQPEGPILCINNCGFFGSAATMNMCSKCHKEMIMKEEQAKLAASSIDSIVNG
CDGGKEHIVAASGSTAVAVAQVEAKTLVVQPTDVAGTSEEVAVVPKVKEGPNRCATCRKRV
GLTGFNCRCGNMYCALHRYSDKHECQFDYRTAARDAIAKANPVVKAEKLDKI*
>LOC_Os09g31200
MAQRDKKDQEPTELRAPEITLCANSCGFPGNPATQNLCQNCFLAATASTSSPSSLSSPVLDK
QPPRPAAPLVEPQAPLPPPVEEMASALATAPAPVAKTSAVNRCSRCRKRVGLTGFRCRCGH

LFCGEHRYSDRHGCSYDYKSAARDAIARDNPVVRAAKIVRF*
>LOC_Os09g21710
MARRGTEAFPDLGAQCDREDCNQLDFLPFDCDGCGKTFCAEHRTYRDHGCARAADQGR
TVVVCEACGDAIERRAGDGGGDDAAVLEAHARSRRCDPARKRKPRCPVPRCKETLTFSNT
SGCKGCGQKVCLKHRFPADHACAGAGAGAASKAAGAAAAARSAGQCGRDAQKKEGG
GWKLPQSVRNMKIF*
>LOC_Os06g41010
MEHKETGCQQPEGPILCINNCGFFGSAATMNMCSKCHKEMIMKQEQAKLAASSIDSIVNG
GDSGKEPIIAGHAEVAVAQVEVKTLVAQPAEIAGPSEGVTVNPKGREGPNRCSTCRKRVGLT
GFNCRCGNLYCAMHRYSDKHDCQFDYRTAARDAIAKANPVVKAEKLDKI*
>LOC_Os05g23470
MAQESCDLNKDEAEILKPSSSSSPSPSPTTASPSPPTAQMTEPPPPQSTPPTPPAAAAAASAA
AAPQFSAKNCEGILIEVSKKRKLAEATATDANAVVVAAVAEPLSPVLFVNRCNVCRKRVGL
TGFRCRCGELFCPRHRHSETHECSFDYKTAGREEIARANPVIRAAKIIKI*
>LOC_Os07g07400
MAPGNEMQARNGGGAAMCAAGCGFFGSAATDGLCSKCYKQQQPQPRHLIGTAAGDSD
KTSLKVVADLSTLVIKDNSGVGGEGTTVMAPPATVTKAKNRCKACRKKVGLLGFPCRCGG
MFCGAHACAFDYKAAGREAIARHNPLVVAPKINKI*
>LOC_Os07g07370
MAGSKMQAGDGGGAAMCAAGCGFFGSAATGGLCSKCYKEQQPQPRHHISSAPPPGTAT
KWWTRSSPTSRRS*
>LOC_Os01g51990
MVLSGRGMLDGGADDVGLWLGMQLPTTSIVDSPGKRSLCVRASDGAGERDAGRAAAAP
VRERLWLLRLCQHPRPLLQVLPRQPPPDRDVPGAVVIVVHGASRRGTVPEGIPVDEGAMPP
PPPPRAKTKSRCAACGRRVGLMGFECRCGAVFCGAHPLLGQARLWLRLQGRAGRDAIAR
ANPVVSADKVDKL*
>MumusD3Z3C6
MFKADLGRIGIQLHTTYSRRIRKVKVMDNRKEPPFFNEDNVGPFYFKLPFYDTMELFIETLT
GTCFELRVSPFEAVISVKGKIQRLEGIPICQQHLIWNNMELEDDYCLNDYNISEGCTLKLVLA
MRGGPISTRKVPVEDPLRELAEYMDSSRDEVWEKTSCNKQVTFLVYREGDQLNFFRVVDRG
DGTLTPLSESLSGSVYNLYTDEDEEAEPSPSGQQIIENSITMNKMKLLKAKMENMNLSKKPK
KVVKVKPRPPLAPRPTSSSTAAARHRLLRVLPHIGQSCLPSGNAHLPETSRNAGPSPAAQAP
ADRPVSSLRNELLKDDDNWEINMLSHSTSSIRLLPQLTHIELESDKELADSVLHLGSSLSRRT
KHLSGNLLSNNEDDVVLFPRSEECVADELLLPEVGAFAPFAEGTGAEQSSGVEGLGKVTPEF
PLTKGDGGLRAAEQPLSHVARVLSSEPGDNAVLNHREPSSHKNRLLSPLLCAAPVSLHNSL
VKPQRQSKCFESGNPSASTSQNTLRELDIRTIADSSFSRTARFRGVKVDSPGKRSDIISKVEAR
DITEMANKASKEPVGCVNNNGFLASLARSASRDSLQSTHGACRLRSSGIGLSTNFQHFQDE
NIRKSSPQSEPTDFFLSARGIGMSGSNAAAGKRIGESIHHLPPVKAPLQTKKKIMKHCFLCG
KKTGLATSFECRCGNNFCASHRYAEAHGCTYDYKSAGRRYLEEANPVVNAPKLPKI
>MumusO88878
MAQETNQTPGPMLCSTGCGFYGNPRTNGMCSVCYKEHLQRQQNSGRMSPMGTASGSNS
PTSDSASVQRADAGLNNCEGAAGSTSEKSRNVPVAALPVTQQMTEMSISREDKITTPKTEV
SEPVVTQPSPSVSQPSSSQSEEKAPELPKPKKNRCFMCRKKVGLTGFDCRCGNLFCGLHRYS
DKHNCPYDYKAEAAAKIRKENPVVVAEKIQRI

>MumusQ497H0
MGDAGSERSKAPSLPPRCPCGFWGSSKTMNLCSKCFADFQKKQPDDDSTPSTSNSQSDLFS
EETTSDNNNTSVTTPTLSPSQQSLPTELNVTSPSTEECGPCTDTAHVSLITPTKRSCGADSQSE
NEASPVKRPRLVENPERPEESGRSKQKSRRRCFQCQTKLELVQQELGSCRCGYVFCMLHRL
PEQHDCTFDHMGRGREEAIMKMVKLDRKVGRSCQRIGEGCS
>MumusQ8BFR6
MAELDIGQHCQVQHCRQRDFLPFVCDGCSGIFCLEHRSKDSHGCSEVNVVKERPKTDEHK
SYSCSFKGCTDVELVAVICPYCEKNFCLRHRHQSDHDCEKLEVAKPRMAATQKLVRDIVDA
KTGGAASKGRKGAKSSGTAAKVALMKLKMHADGDKSLPQTERTYFQVYLPKGSKEKSKA
MFFCLRWSIGKVVDFAASLANLRNENNKLTAKKLRLCHVPSGEALPLDHTLERWITKEECP
LYNGGNVILEYLNDEEQFLKNVDSYLE
>MumusQ91X58
MEFPDLGAHCSEPSCQRLDFLPLKCDACSGIFCADHVAYAQHHCGSAYQKDIQVPVCPLC
NVPVPVARGEPPDRAVGEHIDRDCRSDPAQQKRKIFTNKCERSGCRQREMMKLTCDRCGR
NFCIKHRHPLDHECSGEGHQTSRAGLAAISRAQGLASTSTAPSPSRTLPSSSSPSRATPQLPTR
TASPVIALQNGLSEDEALQRALELSLAEAKPQVLSSQEEDDLALAQALSASEAEYQQQQAQ
SRSLKPSNCSLC
>MumusQ9DCH6
MAQETNHSQAPMLCSTGCGFYGNPRTNGMCSVCYKEHLQRQNSSNGRISPPAASVSSLSE
SLPVQCADGSVPDAQSALDSTSSSMQPGPVSNQSLLSESVAPSQVDSTSVDKAVSETEDLQG
PRAEGLVPLECDPPSSVSDTTQQPSEEQSKSLEKPKQKKNRCFMCRKKVGLTGFECRCGNV
YCGVHRYSDVHNCSYNYKADAAEKIRKENPVVVGEKIQKI
>MumusQ9JII7
MEFPDLGKHCSEPTCKQLDFLPITCDACKQDFCKDHFSYVGHKCPFAFKKDVQVPVCPLC
NAPIPVKRGEIPDVVVGEHMDRDCTFHPGRNRNKVFTHRCSKEGCRKKEMLQLACAQCH
GNFCIQHRHPLDHNCQAGSSSASRGRTSTSRAAEQKPSGVSWLAQRLRRTVK
>MumusB2RUR8
MTLDMDAVLSDFVRSTGAEPGLARDLLEGKNWDVSAALSDFEQLRQVHAGNLSPPFSGGS
TCPKTPEKGGSDREPTRPSRPILQRQDDVIQEKRLSRGISHASSSIVSLARSHVSSNGGGGGSS
EHPLEMPICAFQLPDLTVYKEDFRSFIERDLIEQSMLVALEQAGRLNWWVSMDSTCQRLLPL
ATTGDGNCLLHAASLGMWGFHDRDLVLRKALYALMEKGVEKEALRRRWRWQQTQQNK
ESGLVYTEDEWQKEWNELIKLASSEPRMHLGSNGASGGGVESSEEPVYESLEEFHVFVLAH
VLKRPIVVVADTMLRDSGGEAFAPIPFGGIYLPLEVPASQCHRSPLVLAYDQAHFSALVSME
QKESAKEQAVIPLTDSEHKLLPLHFAVDPGKGWEWGKDDNDNVRLASIILSLEVKLHLLHS
YMNVKWIPLSSDSQAPLAQPESPTASAGDEPRSTPESGESDKESVGSSSLGNEGSRRKEKSKR
DREKDKKRADSVANKLGSFGKTLGSKLKKNMGGLMHSKGPKPGGLGSGSGISSGTETLEK
KKKNNTLKSWKGGKEEAAGDGPVSEKPPSESVGNGGSKYSQEVMQSLSTMRIAMQGEGK
YIFVGTLKMGHRHQYQEEMIQRYLADAEERFLAEQKQKEVERKIMNGGLVSGPPPAKKPE
PDGGEDQPSDSPAEPKAMAFSTAYPGGFTIPRPSGGGVHCQEPRRQLAGGPCVGGLPSYAT
FPRQYPGRPYPHQDNIPALEPGKDGVHRGALLPPQFRVADSYSNGYREPPEPDGWAGAPR
GLPPTQTKCKQPNCSFYGHPETNNLCSCCYREELRRREREPGGELLAHRF
>MumusQ60769
MAEQLLPQALYLSNMRKAVKIRERTPEDIFKPTNGIIYHFKTMHRYTLEMFRTCQFCPQFRE
IIHKALIDRSVQASLESQKKLNWCREVRKLVALKTNGDGNCLMHAACQYMWGVQDTDL

VLRKALCSTLKETDTRNFKFRWQLESLKSQEFVETGLCYDTRNWNDEWDNLVKMASADT
PAARSGLQYNSLEEIHIFVLSNILRRPIIVISDKMLRSLESGSNFAPLKVGGIYLPLHWPAQECY
RYPIVLGYDSQHFVPLVTLKDSGPELRAVPLVNRDRGRFEDLKVHFLTDPENEMKEKLLKEY
LIVMEIPVQGWDHGTTHLINAAKLDEANLPKEINLVDDYFELVQHEYKKWQENSDQARR
AAHAQNPLEPSTPQLSLMDIKCETPNCPFFMSVNTQPLCHECSERRQKNQSKLPKLNSKLG
PEGLPGVGLGSSNWSPEETAGGPHSAPPTAPSLFLFSETTAMKCRSPGCPFTLNVQHNGFCE
RCHARQINASHTADPGKCQACLQDVTRTFNGICSTCFKRTTAEPSSSLTSSIPASCHQRSKSD
PSQLIQSLTPHSCHRTGNVSPSGCLSQAARTPGDRAGTSKCRKAGCMYFGTPENKGFCTLC
FIEYRENKQSVTASEKAGSPAPRFQNNVPCLGRECGTLGSTMFEGYCQKCFIEAQNQRFHE
ARRTEEQLRSSQHRDMPRTTQVASRLKCARASCKNILACRSEELCMECQHLSQRVGSVAHR
GEPTPEEPPKQRCRAPACDHFGNAKCNGYCNECYQFKQMYG
>MumusQ8R554
MVSSLLPNPPSAECWAALLHDPMTLDMDAVLSDFVRSTGAEPGLARDLLEGKNWDLTAA
LSDYEQLRQVHTANLPHVFNEGRCAKQAERELPQPGHKVERPCLQRQDDIAQAEKRLSRG
ISHASSAIVSLARSHVANECNNEQFPLEMPIYTFQLPDLSVYSEDFRSFIERDLIEQATMVALE
QAGRLNWWSTVCTSCKRLLPLATTGDGNCLLHAASLGMWGFHDRDLVLRKALYTMMRT
GAEREALKRRWRWQQTQQNKEEEWEREWTELLKLASSEPRTHFSKNGSGTGGGVDNSED
PVYESLEEFHVFVLAHILRRPIVVVADTMLRDSGGEAFAPIPFGGIYLPLEVPPNRCHCSPLVL
AYDQAHFSALVSMEQRDQQREQAVIPLTDSEHKLLPLHFAVDPGKDWEWGKDDNDNARL
ANLILSLEAKLNLLHSYMNVTWIRIPSETRAPLAQPESPTASAGEDVQSLAESLDSDRDSVCS
NSNSNNGKNGKDKEKEKQRKDKDKTRADSVANKLGSFSKTLGIKLKKNMGGLGGLVHG
KMGRANSANGKNGDSAERNKEKKSKSRKGSKEESGASASTSPSEKTTPSPTDKASGASPAD
KGSGSRGDAWKYSTDVKLSLNILRAAMQGERKFIFAGLLLTSHRHQFHEEMIGYYLTSAQE
RFSAEQEQRRRDAAAAAAATATATVKRPARRPEAEGAPGPERASPGPTAAQPTQLVLKL
KERPSPGTGASARAARAAGGAASPGPGGGARRAAPGTGGPTPGRSPPAPARQSVIHVQAA
ARDEACAPTVGALRPCATYPQQNRSLWSQSYSPARSALRTVNTVESLAPGGADAPGPAEH
KSQTYSNGFGAARDGLEFADADAPAARSNAECGRGGPGPAQRRCQRENCAFYGRAETEH
FCSYCYREELRRRREARAARP
>MumusQ9JM13
MSLKSERRGIHVDQSELLCKKGCGYYGNPAWQGFCSKCWREEYHKARQRQIQEDWELAE
RLQREEEEAFASSQSSQGAQSLTFSKFEEKKTNEKTRKVTTVKKFFSASSRAGSKKEIQEAKA
PSPSINRQTSIETDRVTKEFIDFLKTFHKTGQEVYKQTKMFLEAMPYKRDLSIEEQSECTQDF
YQNVAERMQTRGKVPPEKVEKIMDQIEKHIMTRLYKFVFCPETTDDEKKDLAIQKRIRALH
WVTPQMLCVPVNEEIPEVSDMVVKAITDIIEMDSKRVPRDKLACITRCSKHIFNAIKITKNEP
ASADDFLPTLIYIVLKGNPPRLQSNIQYITRFCNPSRLMTGEDGYYFTNLCCAVAFIEKLDAQ
SLNLSQEDFDRYMSGQTSPRKQESESWPPEACLGVKQMYKNLDLLSQLNERQERIMNEAK
KLEKDLIDWTDGIAKEVQDIVEKYPLEIKPPNQPLAAIDSENVENDKLPPPLQPQVYAG
>Mipus126457
MAERARASEGDALSANLCEAGCGFFGNQNTANMCSVCYSLRTSCTGDNSEIRTNLDTSNS
RIVQPIVTPILETDDDSELDKIDVGPFAVGEPEIKELNKRDEQCLLNRCYTCNKRTGFTGFRC
RCEYIFCSSHRHSNKHNCTFDYKALGRDAVAKANPAVIAEKLHKI*
>Metru3g028010
MAQRTENEETEFKVVSETLQQTTTIINLCIKNCGVVGNPSTNNMCQNCFTASTTILPPSSSRS
VRSPKRSRQESSSSSEEEGSTDHDLVDEKTVSEVKRVVSRCSGCRRKVGLAGFRCRCGELFCA

DHRYSDRHDCGYDYKKVGREEIARENPVIRAAKIVKV*
>Metru3g025570
MNAKTGPSRCITCRKRVDLTGFSCKCGNLFCSMHRYSDKHDCPFNYKFGPECTQAVCLNR
RPMAGYFERFDGSEEDREQEIRADFAAGDARSRYHFGEDSFAEENPITWIP*
>Metru2g086190
MAEEHRLLPPQLCVNNCGFFGSNTTENLCSRCYRDLQLKEQQASSTKFVLNQSIAASSPAV
VVFEPSSSLPDPVGPANVVVVEKPLQHNRCMTCKKRVGLTGFKCKCGRMLCGIHRYPEQH
ACEFDFKGLGKEQIEKANPVVKGEKLEKI*
>Metru2g098160
MDHDQTGCEAAPEGPMLCINNCGFFGSAATMNMCSKCHKDMMLKQEQATLAASSIGNI
MNGSSSSSGIEPAITANVEISVDPVEPKIISAEPLVASGSEESLEKKPKDGPKRCSNCNKRVGLT
GFNCRCGNLYCAVHRYSDKHDCPFDYRTAGRDAIAKANPVVKAEKLDKI*
>Metru2g054650
MDPPPLCLNNCSSAIDKFLCPKCSDDCLTLSEIEGLILGSSPSQNSSILEIDSITVTDTTGKKNN
NRCKTCNKRIGLTGFECRCGDVFCGRHRYPETHSCNVDLKSIGRQILAKQNPKCVLNKLEF
RV*
>Metru8g036980
MAHKITKKEETEFQVPETITPPCINPTQTTTTPTTTTTTTLTEPSRFFEDKSSKARSATSSFSQK
RSHPSNDDSNVQPQTTSSEAKRAVNRCSGCRKRVGLTGFRCRCGDLFCSEHRYSDRHDCSY
DYKAAGRESIARENPVVKAAKIVKL*
>Metru7g091810
MGTPEFPDLGKHCSVSDCRLIDFLPFTCDRCNQVYCLGHRSYIEHNCTKPNKQDVTVVICP
LCAKGVRLIPDQDPNIVWEHHVNTDCDPSNYEKATKKKKCPVPGCRETLVFSNTIKCRDC
EVDHCLKHRFGPDHKCPGPKKLETSFSFMSLWNMSSGKEVSKPNLSSTTSSKWTSSLLDMA
SKLSGIGQSSGSGTSNNQLEKCPLCDAKFSSAASLVDHAKKVHQRSGGNQYGAKKVSISAC
PKCSKGFLDPVSLVEHVERDHGGCS*
>Metru7g114920
MVPSLCANGCGYYGLPSNKNLCSKCYNVYLKENIVLESSSSCPSKNPSINDICDAVAAISLID
SDNMKEKKTRCKSCNKKVGLTGFKCRCGDVFCGMHRYPEEHTCKVDLKKIGRQILEKQN
PLCMGDKLKYRI*
>Metru7g104320
MAEEHRCETPEGHRLCANNCGFFGSSATMNLCSKCYRDIHLKEQEQAKTKSTIETALSSAS
ASTAVVVAASPVAEIESLPQPQPPALTVPSIVPEASDNSSGPVQSNRCGTCRKRTGLTGFKCR
CGITFCGSHRYPEKHECGFDFKAVGREEIAKANPVIKADKLRRI*
>Metru7g092400
MKFKYKQNKRWKQSVQREKEEPFQYNRIKTLLSLCTFVLFIILLLFDFLDFIVIIIIYTVLSLRFE
RTVLQCQDRLTLPPRWSYCRRVMESHDEMGCQAPERPILCVNNCGFFGREATMNMCSKC
YKDTLLKQEQEKLVATSVENIVNGNSSSNGKLAVTASAVDVRVESVELNTVSPEVPENPISN
ESVEMKAKTGPSRCATCRKRVGLTGFSCKCGNLFCSMHRYSDKHDCPFDYRTAGQKAIAE
SNPVIKADKLDKI*
>Metru0249s0070
MLAGDHKIMDSHDETGCQTPELPILCVNNCGFFGRAATMNMCSKCYKDTQLMQEQEKL
AAASVENLVSGGSMKQVVTDGAVNVQIENVEVKTVSAEISGDSSSSENLETKVKTGPSRCA
TCRKRVGLTGFTCKCGNLFCAMHRYSDKHDCPFDYQSVGRDAIAKSNPVIKADKLDKF*

>Metru1g100773
MVPTLCLNGCGFYGSPSKNNLCSKCYNDYLKENIKKSNEESFVHESSSTCPSKNPINDFCDV
VEAISLIDNENMKKKKNRCKSCNKKVGPLGFECRCGDVFCGMHRYPEEHDCTVNLKEIGR
QILDKQNPLCMGDKLEHRI*
>Metru1g060380
MAEEQPCQAPEGHRLCTNNCGFLGNPATMNLCSKCYGDASTKSTIENTLSSSSSVTASPASP
SHSTSEPIVQFINPMVTSSVVITNSVSFLVQSNRCFTCRKRVGLTGFKCRCGSTFCGSHRYPER
HGCGFDFKMVGRKEIAQANPLIKAEKLRRI*
>Metru6g008210
MASGGTEAFPDLGKHCQHVDCHQLDFLPFTCDGCKQVFCVEHRSYKSHECPKPDHNSRK
VVVCEECSMSMEIRGNMGENEEAILKKHRSSGKCDPSKKKKPTCPVKRCKEILTFSNTSTCK
TCHIKVCLKHRFSADHACSKGDSSSLTTTAGNGRWNNRFMAALASRNGQDCGKKSGSRS
TTSPPSNPSVKAC*
>Metru0100s0160
MGTPEFPDLGKHCADSDCKLVDFLPFTCDRCYQVYCLEHRSYIKHRCTKADKQDVTVVIC
PLCAKGVRLVPEQDPNITWENHVNTDCDPSNYEKVTKKKKCPAAGCKEILVFSNTIKCKD
CTIDHCLKHRFGPDHKCPGPKKVETNFPFMNLMNRSRKKESKTNSSSTSSSSKWTTSFLNA
ASNIRASAEAGMSKLSGEINQAWGTSSDGGRKSNDNGQVEQCPQCGAKFSSITTLINHVQ
KVHERSGSRSAANVTIDACPKCSKGFTDPVSLVEHVEREHGGTSRR*
>Metru4g053440
MAEEHRCQAAQRLCANNCGFFGSPAMQDLCSKCYRDLQMKEQRSSSAKLVLNQTLIPQQ
SNSSSLDTGIIHPSSTSPSVMIVSSSTPTVELVAAAAGPSEAEPPKVQPNRCGTCRRRVGLTGF
KCRCGLTLCGTHRYPEQHGCGFDFKGMGREEIKKANPVVKGEKLNKI*
>Metru4g065570
MNMCSKCHKDMMLKQEQAQLAASSLGNIMNGSTSNTEKEPVVTATSVDIPAISVEPKTAS
VDIPESDDPKPKDGPKRCSNCNKRVGLTGFNCRCGNLFCAVHRYSDKHDCPFDYRTSARD
AIAKANPVVKAEKLDKI*
>Mapol0020s0114
MAQESWKRDKEETGCQPPEGPVLCTNNCGFFGSATTSNLCSKCYRDLVLKQAKATSAKAA
AEKAFLTTAPNDFSLERTDQSARQALETLAAVAPSASEPSSGGGTSSSTAADEQEPPRQQAP
NRCFSCKKRVGLTGFKCRCGNTFCSLHRYSDKHSCSFDYKTAGRDAIAKANPVVKADKID
KI*
>Mapol0054s0070
MSGGTEAFPTLGSHCAEEYCHQLDFLPFTCDSCSKVYCLEHRSYKAHECSKVNLKDNVVIV
CPTCTRTVKKVAGESEAVTLKKHNFDGSCSSAGRPAPKARCPVPRCREELLVLNTYSCKSCG
STVCLKHRYPTDHACTTMKNGKFLGALAKRNTSECGSSANSPVNISKGFKTLKDMITRRFD
NLSLQVH*
>Mapol0002s0069
MARESMKALEETGCQPPEGPILCAKNCGFYGTADAMNLCSKCYQMLAAPAASNATPASD
TDMLRSESESTTQFTRQNNEHSGCATEAPPVGETSNATTSGGSSSTATAVVADDPTKRSSHG
PKRCFSCRKRVGLTGFDCKCGNLYCSLHRYSDKHNCTFDYKTAGRDAIAQANPVVKADKI
EKV*
>MDP0000661416
MAEEHRCEAPEGHHLCANNCGFFGSPATMNLCSKCYRDFCLKEQQEASIKSTVEASLSAS

AASAAAASPPCSPPSTSLPSSAAAIETQCQPPPPALTLPEVVGDIIKDPAGDLRAREVAEVVS
QPNRCTVCRKRVGLTGFKCRCGTTFCGVHRYPEKHACSFDFKTLGREEIARSNPLVIAEKLE
KI*
>MDP0000683912
MEHEETGCQAAPEGPILCVNNCGFFGSAATMNMCSKCHKDMMLKQEQAKLAASSFGSIV
NGTSSINANEPVIAAPPVDIQSQPVAPQTISSQPSFSFGSGSSGEPKPEGPKRCSSCNKRVGLT
GFNCRCGHLFCAVHRYSDKHDCPYDYRTAGRDAIAKANPVVKAEKLDKI*
>MDP0000296953
MAWLSSAACDPWKWGGCCNCVHLCVLETIVGHGGLTTNEPKIEIVMAEEHRCQAQQLC
VNNCGFFGSPTTQNLCSKCYRDLQLKEQQAVALNQTLISSSSFASPSSSSSPSPSRPFSSPLISVS
PAHKARVERVVEAKEEEEKEAAPSAGAQANRCTTCRRRVGLTGFKCRCGMTFCGTHRYPE
QHACGFDFRGMGKEQIAKANPVVKAEKLHKI
>MDP0000874708
MEHNETGCQAPPEAPKLCANNCGFFGSPATMNLCSKCHKDLVLKQEQAKVVAASIDSAV
NGSPSESGKGPVATAAVDVQAGPADVMLISTQASSTPSLNIKREEKVKETPTRCGTCRKRVG
LTGFSCRCGDLFCAVHRYSDKHNCPHDYRTAAQDAIAKANPVVKAEKLDKI*
>MDP0000362677
MKKMAQRTEKEETEFKVPETLTHCVNNCGVTGNPSTNNMCQKCXNAASAAAAAATSSSS
SAAILKFSAEKSPRSSSSFSFEAAAETCRKTTASEIARSDETPNRRVVNRCSGCRRKVGLTGFR
CRCGELFCSEHRYSDRHVCSYDYKAAGREAIARENPVVKAAKIVRVX
>MDP0000516205
MAEEHRCEAPEGHHLCANNCGFFGSPATMNLCSKCYRDFCLKEQQQASIKSTVEASLSAS
AADAASSSLSAPSSPPSTSLPASPAAIETLCQPPPPALTLPEVAGDIIGEPAEVVRAPEVATVVS
QPNRCTVCRKRVGLTGFKCRCGTTFCGVHRYPEKHACSFDFKTLGREEIARSNPLVIAEKLE
KI*
>MDP0000292844
MVKYILRVLASREWCRFQYCRYLVGGDVYLELCYCIILKDLANYNAGNYKKMEHNETGCQ
APPEAPKLCANNCGFFGSPATMNLCSKCHKDLVLKQEQAKVVAASIDSVVNGSPTESSKGP
VATAAVDVQAGSADVMLISTQASSTSLNIKSEEKVKETPTRCGTCRKRVGLTGFSCRCGDIF
CAVHRYSDKHKCPYDYRTAAQDAIAKANPVVKADKLDKI
>MDP0000141121
MTGGTEAFPDLGRHCQLSDCHQLDFLPFXCDGCHRVFCVEHRSYKSHECPKSDHNSRKVV
VCEICSTSIETTGRDGEQDQKLLLEKHAKSGNCDPRKKKKPTCPVRRCKEILTFSNTSTCKTC
KIKVCLKHRFPADHVCQKQTAAQPSLVGKPVSWNDKFMAAFALREGKECGKSARDSKSS
ASSAPSVRAY
>MDP0000652898
MGTPEFPDLGRHCYVAECQQIDFLPFTCDSCHQVFCLEHRSYIKHNCPKGDRKNVTVVICP
LCAKGVHLIPDEDPNITWERHVNTDCDPSNYEKATKKKKCPVPGCKEXLTFSNTIKCRDCT
VDHCLKHRFGLDHKCPGPKKPEAGFPFLGYLSRSRKEVSKPNHAPAASSXNWSSFLTAASS
FRASAEASVAKLSSELSQKWQIAKDGTGQSSSSSGSRNGQGEVCPQCGAKFSSVTTLVDHVE
KVHEKGGNRAAAVKKVTIDACPKCSKGFRDPVALVEHVERDHGGTSRA*
>MDP0000211516
MXGGTEAFPDLGRHCQLSDCHQLDFLPFQCDGCRKVFCVEHRSYKSHECPKSDHNSRKV
VVCEICSASVETTGCDGEQDHKLLLQKHAKSGNCDPKNKKKPTCPVRRCKETLTFSNTSTC

KTCQIKVCLKHRFPADHVCRKQTAAQPSLLAGNGVSWNDRFMAAFASRKGKECGKSERD
SKSSASSGPSVRAY
>MDP0000305771
MIEVKKERPLGVLPRIPDAEVGYTFKKLRPEVFKENNTFTPPYNCPKSVILRWLQRLCLFFSL
GEKRKLGRPWPWDSRVFGVSLCIRGSQNPIQASDNPIPIPIPASPNKSRDQIIRSILYISTHFVD
WRRASTAQRAKFGWELRNSQIWVFCLEHRSYIKHNCPNGDRNNVTVVICPLCAKGVHLIP
DEDPNITWERHVNTDCDPSNYEKATKKKKCPIPGCKEILTFSNTIKCRDCMVDXCLKHRFG
PDHKCPGPKKPEAGFPFMGYLSRSRKEVSKPSHAPAASSPKWGSFLTAASSFRASAEASVAK
LSSELSQKWQIAKDGTGPSSSSSGSRNGQVEVCPQCGAKFSSVTTLVDHVEKVHEKSGNRA
AGVKKVTIDACPKCSKGFRDPVALVEHVERDHGGKERPISEVPIFPSCQIVCILYSKMSAVSG
VISRQVLPACGSLCFFCPALRARSRQPVKRYKKLIADIFPRNQEEGPNDRKIGKLCEYVAKNP
LRIPKTDGTYTFNLEGFIPKLCQIAQEPGEDERASNLRSAALQALSSMVWFMGEKSHISVKF
DSIVAVVLENYGGSNKTTENLEGSKRWVQEVQSNEGHGSSSPDVTIRLTSWSTLVDDKGEL
NVTVEDAKNPCFWSRVCLHNMAKLAKEATTIRRVLESLFRYFDTGNLWSPKYGLAIPVLK
DIQVLMDDCGQSTHILLSILIKHLDHKNVLKQPNMQLEIAEVTTSLSQLAKVEPSVAIIGAVS
DAMRHLRKSIHCSLDDANLGTDVTKWNRSFREVVDKCLVQLCYKVGEPGPILDAMAVML
ENISTITVIARNTISAVYRTAQIVASLPNLSYQNKASSITYVLAFPEALFHQLLPAMVHPDHET
RVGAHRIFSVVLVPSSVCPTRSSPNTESKKALNFPRTLSRTVSVFSSSAALFEKLRREKISSRESI
CEEDNENTDNEGEQRDTNNGILSRLRSSYSRTYSIKSSPAPSSTKENSVNSSTKEPEANSLRLS
SHQITLLLLSIWAQSISPGNMPENYEAIAHTLSLVVLFSRAKNSRIEVLVRSFQLAFSLRNISLN
EGGLLPPSRRRSLFTLATSMILFLSKAYNIVSLVCRAKAVLVDKIVDPFLHLVEDRKLQAVKT
GPNHPRHLYGSEEDDNMALKSLSEINITEEQTKEFFASEVVKSLERLLDAEMSTIREELLSEFL
PDDVCPLGAQLCMDAPQKLYQVDSRDTKSMKEDAPIFSLDDDYFQGSFDSQKNNLDFSAE
SHNLLSVSQLIESVLETAHQVGRVSVSNAPDVPYKEMAGQCEALLLGKQQKMSNLMSSQP
KQEYLMNQSLQNHSDDAKWMTSDSHRSGNPFVDDTANSYKPSPSTGHAPMMCVTEYQH
HPHSFRLPASSPYDNFLKAAECGLEVQLKPNDSMQCRDCGYHILYKKRARRNANSRCEAP
YTMLHPRRLGSCEVGDGKLKRRTFGILCELLRTRGRLRMYGSVSIEDPIRAAPMDPPPPEAV
YYICGDCGMEVQLKSNDVIQCRECGYRILYKKRTRRSKSSLXLYDXIQRLIMTNGCFCSCSVR
GALKNPGGALSMATDDVNDRWRLLNSAMFYTTCTVLTRNSPIGQGV
>MDP0000164222
MEHEETGCQAAPEGPILCVNNCGFFGSAATMNMCSKCHKDMMLKQEQAKLAASSFGSIV
NGTSSIXANEPVVAAATVDVQPHPVEPKTLSSQPSFSFGSGSSGEPXPEGPKRCNTCNKRVG
XTGFNCRCGHXFCAVHRYSDKHDCPYDYRTAGRDAIAKANPVVKAEKLDKI
>MDP0000293524
MEPPPLPQSSKPNPTSSFSSVDSEVLLKSIDQVRKRLNYETGVAEYRNYKLMKAEKELMDLN
GEANEAFYCASKQKTNADTSGERITFLNFELFQKRRGHIESYERSREGNAEIKEERNKDFED
SRRLVKERNHVNLNGIIWHFIVVLANCCLNQRAELVLYIKHVLDALSKKGGIFVMDLYGGT
SSDCKIRLQRTFANFTVQNFYSLSTCIFMQLEAMEDSKEIRRAEGLGVGRDIKYEEAKHFQQ
HGSWNADIVGVALLHAPGKLCVERIEIVMAEEHRCQPPQLCVNNCGFFGTQGTTGARAQ
PNFDLLLFFSLFLMSAFISAGLRLARARGAGGACDRSEGRREGGGAEHDVPAAPSAGAHA
NKCMTCRRRVGLTGFQCRCGMTFCGTHRYPEQHACGFDFRGMGKEQIAKANPVVKADK
LQKI
>MDP0000286185
MCPSSPDQTPNPRTVHSPTGTCTVKWQHRNYVVVEGRLHRYHRLSGKRSXAKRKEKMES

HDETGCQAPDRPILCVNNCGFFGRAATMNMCSKCYKDTLLKQEQANLAASSIDSIVNGG
GSSSSSNIFIDPVVASVVDVQAVRVETSVVSTEAYIESSPSMKIEMKENKGPSRCTTCRKRVGL
TGFNCKCGNTFCASHRYSDKHDCPFDYRTAGQDAIAKANPIVKADKLDKI
>MDP0000294781
MCPSSPDQTPNPRTVHSPTGTCTVKWQHRNYVVVEGRLHRYHRLSGKRSXAKRKEKMES
HDETGCQAPDRPILCVNNCGFFGRAATMNMCSKCYKDTLLKQEQANLAASSIDSIVNGG
GSSSSSNIFIDPVVAXVVDVQAVRVETSVVSTEAYIESSPSMKIEMKENKGPSRCTTCRKRVGL
TGFNCKCGNTFCASHRYSDKHDCPFDYRTAGQDAIAKANPIVKADKLDKI
>MDP0000362676
MKKMAQRTEKEETEFKVPETLTHCVNNCGVTGNPSTNNMCQKCXNAASAAAAAATSSSS
SAAILKFSAEKSPRSSSSFSFEAAAETCRKTTASEIARSDETPNRRVVNRCSGCRRKVGLTGFR
CRCGELFCSEHRYSDRHVCSYDYKAAGREAIARENPVVKAAKIVRVX
>MDP0000086327
MESHDETGCQAPDRPILCVNNCGFFGRVATMNMCSXCYKDTLLKQDQAXLAASSIDSIVN
GVXXSSNIVIDPVVASVVDVQXVQVGTSIVXTEPSSDSSSSMKIEVKEKKGPSKCTTCRKRVG
LTGFNCKCGNTXCASHRYSDKHDCPFDYKTAGQDAIAKANPIVKADKLDKI
>MDP0000122842
MEPPMCASGCGFYGTVENKNMCSKCYKDHLKHETMNAASADVTSKEKLNLGSFISGISSS
SYFRTSSDTSLVSEDHNFGNNNMGTSSVGVIKKNRCQSCSRKVGVLGFQCRCGGVFCGTH
RYPEEHSCDVDLKQAGRDVLAKKNPLCKGDKLEWRI
>MDP0000139359
MAEEHRCQAQQLCVNNCGFFGSPTTQNLCSKCYRDLQLKEQQAVALNQTLISSSSFASPSS
SSSPSPSRPFSSPLISVSPAHKARVERVVEAKEEEEKEAAPSAGAQANRCTTCRRRVGLTGFK
CRCGMTFCGTHRYPEQHACGFDFRGMGKEQIAKANPVVKAEKLHKI
>MDP0000133254
MDSRNDTNMEPPLCAKGCGFFGSVTNMNMCSNCYRQYLKEEQFAKPAAMVGLASVDNT
LSDSSSATAAVISSLPSQSSQGSSDLSQKKRCLSCKKRVGPTGFECRCGGVFCGKHRYPEEHS
CSVDYKKTGQELLTKQNPLCKGDKLHWRV
>MDP0000588934
MESQKKMTERPCCANGCDFYGSVETKNLCSRCYXDYLKQESRENMRAESAMVAXMNNL
DRGSVAGRINPLPSLKASNSVSPSVAVAGCSKSSSGSTSVKNRCESCNRKVGVLGFSCRCGG
VFCGTHRYPEKHCCHVDFKMAGRDVLAKQNPLCKGDKLECRI*
>MDP0000707978
MAEEHRCQAQQLCVNNCGFFGSPTTQNLCSKCYRDLQLKEQQAVALNQTLISSSSFASPSS
SSSPSPSRPFSSPLISVSPAHKARVERVVEAKEEEEKEAAPSAGAQANRCTTCRRRVGLTGFK
CRCGMTFCGTHRYPEQHACGFDFRGMGKEQIAKANPVVKAEKLHKI*
>MDP0000494946
MESRKDINMEPPRCAKGCGFFGSVTNMNMCSKCYRECLKEEQFAKPAAMVGLASVDKPLI
VSNSTATAAVISSLPSQSSQGSSDSSEKKRCLSCKKRVGPTGFECRCGGVFCGKHRYPEEHSC
CVDYKKTGQDLLTKQNPLCNGDKLDWRV*
>MDP0000231017
MEHEETGCQAAPEGPILCVNNCGFFGSAATMNMCSKCHKDMMLKQEQAKLAASSFGSIV
NGTSSINANEPVIAAPPVDIQSQPVAPQTISSQPSFSFGSGSSGEPKPEGPKRCSSCNKRVGLT
GFNCRCGHLFCAVHRYSDKHDCPYDYRTAGRDAIAKANPVVKAEKLDKI

>MDP0000506127
MAEEHRCEAPEGHHLCANNCGFFGSPATMNLCSKCYRDFCLKEQQQASIKSTVEASLSAS
AADAASSSLSAPSSPPSTSLPASPAAIETLCQPPPPALTLPEVAGDIIGEPAEVVRAPEVATVVS
QPNRCTVCRKRVGLTGFKCRCGTTFCGVHRYPEKHACSFDFKTLGREEIARSNPLVIAEKLE
KI*
>MDP0000263150
MAQRAEKEETEFKVPETLTHCVNNCGVTGNPSTNNMCQKCFNAASAAATTSSSSSSAAIL
KFSAEKSPRSTSSFSFEAPVETFRKTTASEIARSDESPNRRVVNRCSGCRRKVGLTGFRCRCGE
LFCSEHRYSDRHVCSYDYKAAGREAIARENPVVKAAKIXNEHSRKRQGVFRHLIFATGNSV
ADGGKQWGPWNGHEMRFYIINFIAIISIDFNISLACWREGLCEADVCAVLTVKAVKRWK
>MDP0000237812
MEPPMCASGCGFYGTVENKNMCSKCYKDHLKHETMNAASADVTSKEKLNLGSFISGISSS
SYFRTSSDTSLVSEDHNFGNNNMGTSSVGVIKKNRCQSCSRKVGVLGFQCRCGGVFCGTH
RYPEEHSCDVDLKQAGRDVLAKKNPLCKGDKLEWRI
>MDP0000853499
MTGGTEAFPDLGRHCQLSDCHQLDFLPFHCDGCHRVFCVEHRSYKSHECPKSDHNSRKV
VVCEICSTSIETTGRDGEQDQKLLLEKHAKSGNCDPRKKKKPTCPVRRCKEILTFSNTSTCKT
CKIKVCLKHRFPADHVCQKQTAAQPSLVGKPVSWNDKFMAAFALREGKECGKSARDSKSS
ASSAPSVRAY*
>MDP0000543745
MTQRAEKEETEFKVLETLTHCVNNYDVTGNPSTNNMCQKCFNAATAAATTSSSSSSVTILK
FSAEKSPRSTSSFSFEAPAETFKKTKASEIARSDESPNRCVVNQCFECRRKVGLTGFRFYLAILS
IISRYISQIMH*
>MDP0000284856
MAEEHRCEAPEGHHLCANNCGFFGSPATMNLCSKCYRDFCLKEQQEASIKSTVEASLSAS
AASAAAASPPCSPPSTSLPSSAAAIETQCQPPPPALTLPEVVGDIIKDPAGDLRAREVAEVVS
QPNRCTVCRKRVGLTGFKCRCGTTFCGVHSTPRSTR
>MDP0000165407
MAEEHRCEAPEGHHLCANNCGFFGSPATMNLCSKCYRDFCLKEQQXASIKSTEMLRVDRV
QVQVRDHVLRGSQVPREARVLVRFQDPRKGGDRQEQPVGHSREAREDLIRSVGSNTFVKI
QIADRRVSINQAVRGTDDMIRTFTLSPIRQMLENSSSFGSTSSSPSLANLPSIRVHVEDGIGGG
GEDLGRVQAGEMGRSQSRVSGLGLF
>MDP0000316313
MAQRAEKEETEFKVPETLTHYVNNCGVTDNPSTNNLCQKCFNTATTSSSFSSAAIXKLSAE
KSPISTSSFSFEALAETFRKTTASEIARSDESLNRRVVNRCSECRRKVNYTELSLGNKSPTFPLP
FAPKIRWKLASNWVKCTDGFRGCTVAINRFYKHHC
>Gorai009G404700
MAEEHRCQAPEGHRLCVNNCGFFGSSATMNLCSKCYRDLCLKEQEASSIKSALSSSPSSSST
VVESISQVPLLALAEVNRESAVPEIAPAAEQLSQQQPNRCMVCRKRVGLTGFRCKCGVTFC
GSHRYPENHGCTFDFKKVGREEIARANPLVKAEKLEKI*
>Gorai009G086500
MAEEHRCQAPQLCANNCGFFGSPTTQNLCSKCYRDLQLKEQQSSSAKQAFNHTLVPSSSSL
PSSSSARSSFSASLPAKEEPSAGTKETKVVEEEEVQVTPNRCLSCKKRVGLTGFKCRCGMVFC
GIHRYPEQHACAFDFKGMGKQQIAKANPLVKGEKLQKI*

>Gorai004G173900
MAQRTEKEETEFKVPETLTLCVNNCGVTGNPATNNMCQKCFSATTAATSSSSSSTNNTATS
ATDDKSSRSTPTRSQDNRSDSAPPTTAATTATATTNSPMTASNRSGYDTAEKKSVNRCSG
CRKRVGLTGFRCRCGELFCSDHRYSDRHDCSYDYKAAGREAIARENPVVKAAKIIRV*
>Gorai004G036200
MAQRTEKEETEFKVPETLTLCVNDCGVIGNPATNNMCQNCFNATTPKSTAAVVSSSTGGAS
SGGVSILNQRSTTSRSINKRSDLSPPKTTTFVRSSGSRYDPEPGTEKKVVNRCSGCRKRVGLTG
FRCRCGELFCADHRYSDRHDCSYDYKTVGREAIARENPVVKAAKIIKV*
>Gorai007G358100
MESHDETGCQAPEGPILCINNCGFFGSAATMNMCSKCHKAMILKQEQAQLAASSIGIIVN
GSSTGNSKEPAVATALDVQSGNADTKLVSTELPIDPSGTTSCGMKTKEGPNRCTKCCKRVGL
TGFNCRCGNLFCAAHRYSDKHDCPFDYRAAARDAIAKANPVVRAEKLDKI*
>Gorai007G247400
MNDPTSSAAAPGPSDSVVSATLISSPSKINNRCESCNRKLGLMGFTCRCGRVFCQFDRYPLE
HSCNYDFKKAGRQSLAKENPVIRGDKLKSRM*
>Gorai013G258400
MAEEHRCQAPKLCANNCGLIGSPATQNLCSKCHRDLQLKQHRSSSAKHAVNQTSIPSLSSF
PSVSSSSSADKDAGSVAETKAAEVVEVEVRPKRCLSCNKRVGLTGFKCRCGMVFCGIHRYP
EEHGCKFDFKAMGKQQIAQANPVVKAIKLHKI*
>Gorai002G230400
MGTPEFPDLGRHCSVQHCKQIDFLPFTCDRCDLSYCLEHRSYIKHQCSKAGNNDVTVVICP
LCAKGVRLIPNEDPNISWENHVNTECDPSNYDKVTKKKKCPVRRCKEVLTFSNTIKCKDCS
VDHCLKHRFGLDHDCPGPKKPATTSSSFWATSLLNVASSFRENRQVARDGAGLRRSSSGSA
GQMEECPQCRVKFSSVTALVEHVKKVHERNSQSRVLKMSIDVCPKCGRGFRDPVSLVEHVE
KDHGGTSKA*
>Gorai002G229200
MDPLDETGRQASEGPILCVNNCGFFGSAATMNMCSKCHKAMILKQEQAQLVASSIDSIVN
GSTSGNGKEPSVAAALAVQCGNFGSKIESSIDPSHMTFSGMKTKEGPNRCNACHKRVGLTG
FSCKCGNIFCAAHRYSDKHNCPFDYRTAARDAIAKANPVVRAEKLDKI*
>Gorai002G240900
MAEEHRCQAPEGHRLCVNNCGFLGSPATMNLCSKCYRDFRLKEQQQASSAKSSISTSPSSSS
TAVESVSQVPLLTLPQVKGVPPVVSAVAISPVTEQKPQQQQQQQQQQPTRCTVCRKRVGL
TGFKCKCGITFCGSHRYPENHGCTFDFKKIGREEIARANPVVKAEKIVRI*
>Gorai003G167800
MGSEQNEGTSFPPSEPKLCANGCGFFGTAANMNLCSKCYRDLRVGEEQAAKAKAVMEKS
LSIKTKHEPVVVETFKPHVGSSSTSIEQQQPAVVAVNQQPEPKAANRCFICRKKVGLTGFKC
RCENTFCGEHRYPEKHECSFDFKGIGRDAIAKANPVVKADKVERF*
>Gorai008G240900
MGSEQNEGTSFPPSEPKLCANGCGFFGTAANMNLCSKCYRDIRAGEEQAAKAKAAMEKS
LSVNTKQEDVVDETVKPVLELPHVGSSSTVVEKQPAAIVSDDKPAEPKAANRCFICRKKVG
LTGFKCKCGSTFCGEHRYAEKHDCSFDFKGTGRDAIAKANPVVKADKVERI*
>Gorai001G121500
MNETKPSNSALELKKKKISDMAEEHRCKAPPQCANNCGFFGNPATQNLCSQCYRHLQHL
KEQGSSSAAKQAFNQALLPSFSSSSSFSVSLAVKHEPLAETKEEVVQAEVQVHVQVRPNRC

MTCKKRVGLTGFKCRCGMVFCGTHRYPENHGCSFDFKGMGKQQIAKSNPVVKGEKLQKI
*
>Gorai001G259200
MAGGTEAFPDLGKHCQFSGCYQLDFLPFKCQACHKVFCVEHRSCKSHECPEPEHNSRKVII
CEICSMSIEITGKEDQEKMILEKHEKSGNCDPRKKKKPTCPVKRCKETLTFSNRTICKTCRLE
VCLKHRFPADHACKQASSSTTPAAAAGGSWNEKFLVAFGLRNGKDCGKSGRPSSSTTPFLK
AY*
>Gorai006G139100
MESHDETGCQAPEGPILCVNNCGFFGSAATMNMCSKCHKAMILKQEQVQLAAVSIGSIVS
GCSSGSSKEPAVAAALDVQPVNIESKIVSAEPSIDPSRTTVSELETKKGPNRCSTCHKRVGLTG
FNCRCENLFCAAHRYSDKHECPFDYQAAARNAISKANPVVRAEKLDKI*
>Gorai006G031300
MSSSCHSLFRTILKLPIVVEAFKPHVGSSSTSIEQQLPVDVVVACSQQLEPKAANKCFICRMK
VGLTGFKCRCENTFCGEYWYPKKHECSFDFKGVGCDAIAKANPIAKADKVERF*
>Gorai006G211300
MGSEQNQGTSFPPSEPKLCANGCGFFGTAANMNLCSKCYRDLRAGEEQAAKAKAAMEKS
LSVKPKEDVVVETFKPVEKLPHAGSSSAAVEQPAVALSGDEQPEPKLSSRCFICRKKVGLTGF
KCRCGSTFCGEHRYPEKHECSFDFKGTGRDAIASANPVIKADKLERF*
>Gorai006G156200
MIASLRFISFVHILPSTFSFQDLADLTSSKFNFTRNLADRKILSFLKMAEEHRCQTPEGHRLCV
NNCGFFGSPATMNLCSKCYRDFRLKEQQGATSIKSSLSSSSSSSSVVVESVSQVPLFTLPEFIGE
SPVPAVEVALVAEQRPQQQQPIRCMVCRKRVGLTGFRCKCEITFCGSHRYPENHGCTFDFK
KVGREEIARANPVVKAEKLEKV*
>Gorai006G137400
MGTPQFPDLGKHCSVEDCKQIDFLPFTCDRCHLVYCLEHRSYIKHHCPKADKKDVTVVICP
LCAKGVRLIPDEDPNITWEMHVNTECDPSNYDKVTKKKKCPVPGCREVLTFSNTIKCRDCT
LDHCLKHRFGPDHKCPGPKKSDPGFPFMGLLSRSRKESKTNQAPATSSSKWATSFLNAAST
VRASAEAGMTKLGSEISQKLQIARDGVGLSSSGGGSNGNAGQVEECPQCGAKFSSVTTLVD
HVEKVHERNNQSRVFKMSIDVCPKCSKGFRDPVALVEHVERDHGGTSKA*
>Glmax10G103400
MGTPEFPDLGKHCAVSDCKLIDFLPFTCDCCDQVYCLDHRSYNKHQCTKADKQDVTVVIC
PLCAKGVRLVPDQDPNITWENHVNTECDPSNYEKVTKKKKCPVPGCRVILVFSNTIKCRDC
TVEHCLKHRFGPDHKCPGPTKVESSFSFMNLLNGSKKQESKPKSSATSWSTSFLNAASNVR
ASAEAGVSKLSSWQTARGGVGQSHSSGQVEQCPQCGAKFSSVTTLVDHVQKVHERSRNRS
GAKVTIDVCPKCSRGFRDPVALVEHVERDHGGSSRS*
>Glmax10G070900
MAEEHRCEPPEGHRLCVNNCGFFGSTATMNLCSKCYSAIRLKEQEEASTKSTIETALSSASS
AKPSSSTSPPPSAVDVLMESPPPSAAEVEVAVTVTVAVASSSISINSGSVAQPNRCATCRKRVG
LTGFKCRCGVTFCGAHRYPEEHACGFDFKTVGREEIARANPVIKAEKLRRI*
>Glmax17G136800
MGSSERKPCANNCGFFGTSEKLNLCSKCYKDLCLEEELASMKSVLCSAKPPSPAEPSQGTEG
PSKPANRCGTCNKKVGLTGFVCKCGSTFCGVHRYPEKHKCTYDFKGEAREAIAKANPVVK
GDKIDRF*
>Glmax09G156100

MVPSLCANGCGFYGSSATKNLCSKCYIDYLKENVTKSKECETKNLSDQVFVLDQSSATSCK
PCASEVSNTDDATMVDVSLTDPEGIINNKRKKRCKCCNKKVGLLGFECRCGDVFCGTHRY
PEKHACHVDLKEIGRQGLVKQNPVCIGDKLKHRI*
>Glmax15G033400
MDHDKTGCEAPPEGPILCITNCGFFGSAATMNMCSKCHKDMMLKQEQAGLVASSIGNIM
NGSSSSSGNEPAIATSVDVSVNSIEPKIIPAQPLVASGSEESDEAKPKDGPNRCSNCNKRVGLT
GFNCRCGNLFCSVHRYSDKHNCPFDYHTAARDAIAKANPAVKVEKLDKI*
>Glmax15G272300
MAQKTEKEETDFKVPETITLCVNNCGVTGNPATNNMCQKCFTASTATTSGAGGAGIASPA
TRSGVSARPQKRSFPEEPSPVADPPSSDQTTPSEAKRVVNRCSGCRRKVGLTGFRCRCGELFC
AEHRYSDRHDCSYDYKAAGREAIARENPVIRAAKIVKV*
>Glmax13G069200
MASGGTEAFPDLGKHCQHRDCNQLDFLPFTCDGCQQIFCLEHRSYKSHACLKSDHNSRK
VVVCEACSMSIETTGHVGQDEEAILQKHLKSGNCDPTKKKKPICPVKRCREVLTFSNTSTCK
TCHIKVCLKHRFPADHACSRGASASSSACVSNGLWNNRFLTAFAKRTGQECAKNGATCST
SPPSTPSVKAY*
>Glmax13G341000
MDHDKTGCQAPPEGPILCINNCGFFGSAATLSMCSKCHKDMMLKQEQAKLVASSI

ALL ZmSAPs CDS and protein sequeces
>Zm00001d034389
ATGACGCAGAAGCGGAAGAGCATCGGCCGCGGCGGCGTCGAGGACTGCGGAAGTCC
CGCCCGCGCCGCAGCGTGCACCAGTACCACGGCGGCGTCAACCTCTACCACTGTCGA
GGAGAAGAACACGCCGGCTGTTTTCGAGACAACGCCGCCACTTTGGATGACGGGGCC
GGCAGAGACGAAGAAGCCCAAGATCGCGTCGTCGTCGTCGTCGTCGTCGTCGTCCTC
CTCCTCCTCTCCTGACGGAGGCAGCAATAATGCAGCCGTGGCACAGGCCCAGCCGCA
GCCGCCGCCGGCGAACCGGTGTTCGGCGTGCCGCAAGAAGGTGGGCCTGCTGGGGTT
CCGGTGCTGCTGCGGGAAGACCTTTTGCGGCGCGCACCGCTACGCGGAGAAGCACGC
CTGCGGCTTTGATTACAAGCACGCCGGTCGCGGACGGATCGCCAAGGAGAACCCAAT
CATCGTCGCCGACAAGATCGCCAAAATTTAA
>Zm00001d053671
ATGAACATGTGCTCCAAGTGCCACAAGGAGATGATAATGAAGCAGGAGCAGGCCCAG
TTGGCTGCCTCCTCCATCGATAGCATTGTCAATGGCGGTGATAATGGGAAAGGACCTG
CAATTGCTGCAACTGTAGGTGTGGCAGTTCCTCAAGTCGAGGAGAAGACTATCGCCGT
GCAGCCTATGCATGTCGCTGAAACCAGCGAGGCTGCTGCTGTTATCGCGAAGGCCAA
GGAAGGCCCAAACCGGTGCGCAACCTGTAGGAAGCGTGTTGGGTTGACGGGTTTTAA
CTGCCGATGCGGGAACACGTACTGTTCGATGCACCGCTACTCCGACAAACACGACTG
CCAGTTCGACTATCGAACTGCAGCTAGGGACGCTATCGCCAAGGCCAATCCAGTGGTG
AAGGCGGAGAAGCTTGACAAGATCTGA
>Zm00001d020926
ATGGCGCAGCGCGACAAGAAGGAGGAGCCCACGGAGCTGCGGGCGCCGGAGATCGC
GCTCTGCGCCAACAACTGCGGCTTCCCGGGCAACCCGGCCACGCAGAACCTGTGCCA
GAGCTGCTTCTCGGCGTCGAGGTCGTCGTCGTCGTCTTCGCAGCCGTCCCCTACCTCCT

CTCCCTCAGCTTCCGCGCCGGCCGCCGCGGTGCCGCAGCCGAGGCCGGCGCTCCTGG
ACGCGGCGCTGCAGCTGGCGCCTCCGGCCGCGGCCGCGGGCCAGCCTGTGGAGGCCT
CGGCCAGGACTTCTGCCAACCGGTGCTCCAGCTGCCGGAAGCGCGTGGGGCTGACGG
GGTTCCGGTGCCGCTGCGGCGACCTGTTCTGCGGCGCGCACCGGTACTCGGACCGCC
ACGGCTGCCGCTACGACTACAGGGGCGCCGCCCGGGACGCCATCGCCCGGGAGAAC
CCGGTGGTGCGCGCGGCCAAGATCGTTAGGTTCTGA
>Zm00001d031423
ATGGCTCAGCGCGACAAGAAGGTGGAGGAGCCGACGGAGCTGCGCGCGCCGGAGCT
CACGCTGTGCGCCAACAGCTGCGGCTTCCCGGGCAACCCGGCCACCAACAACCTCTG
CCAGGCCTGCTTCCTGGCCGCCACGGCTTCCTCCTCCGCCTCCGCCTCCGTCTCGCCGC
CGCCGCCCTCCTCCTCCTCCTCGTCGCCGGCGGTGCTCCAGTTCGACGAGCAGCAGCA
GCAGCAAAATCCGAGGCCGCGTGCGCCGGCGGCGTCCGGGCCCACGGAGGAACCGC
CCAGGCCGGCCCGGGCGTCAGCTCCGGCTCCGGCTCCGGCGTCGTCCTCGTCCGTCCG
CCGGTGCCAGACCTGCCGCAAGCGGGTGGGGCTCACGGGCTTCCGCTGCCGCTGCGG
AGACCTCTTCTGCGGCGCGCACCGCTACTCGGACCGCCACGACTGCTGCTTCGACTAC
AGGGCCGCCGGCCGCGACGCCATCGCCAGGGACAACCCCGTCGTGCGCGCCGCCAA
GATCGTTAGGTTCTAG
>Zm00001d015842
ATGGAACACAAGGAGGCGGGCTGCCAGCAGCCGGAGGGCCCAATCCTATGCATCAAT
AACTGCGGCTTCTTCGGCAGTGCTGCGACGATGAACATGTGCTCCAAGTGCCACAAG
GAGATGATAACGAAGCAGGAGCAGGCCCAGCTGGCTGCCTCCTCCATCGATAGCATT
GTCAATGGCGGTGACGGCGGGAAAGGACCTGTAATTGCTGCATCTGTAAATGTGGCA
GTTCCTCAAGTTGAGCAGAAGACTATTGTTGTGCAGCCCATGCTTGTAGCTGAAACCA
GCGAGGCTGCTGCTGTAATCCCCAAGGCCAAGGAAGGCCCAGACCGGTGCGCGGCCT
GCAGGAAGCGTGTTGGGCTGACGGGATTTAGCTGCCGATGCGGGAACATGTACTGTTC
GGTGCACCGCTACTCCGACAAACATGACTGTCAGTTCGACTATCGGACTGCAGCAAG
GGACGCGATTGCCAAGGCCAATCCTGTGGTGAGGGCGGAGAAGCTCGACAAGATCTG
A
>Zm00001d021842
ATGGGCACGCCGGAGTTCCCCAACCTGGGAAAGCACTGCAGCGTCGGCGACTGCAA
CCAGATCGACTTCCTCCCCTTCACCTGCGACCGCTGCGACCATGTCTTTTGCCTTGATC
ACCGAAGTTATACATCACACCAATGCCCAAATGCAAACATGAAAGACGTCACTGTCCT
CATCTGCCCACTCTGTGCTAAAGGTGTTCGCCTCAATCCAAGTGAAGACCCAAATATC
ACCTGGGATACTCATGTTAACACTGATTGTGATCCATCAAATTACCAAAAAGTGATGA
AGAAAAAGAAATGCCCTGTTCCTGGGTGCAGAGAGACACTGACATTTTCCAACACCA
TCAGATGCAAAGATTGCACCAGAGAACACTGCCTAAAGCACAGATTTGGGCCTGATC
ATAAGTGCCCAGGACCAAGAAAAGTGGATTCTGGCTTTCCCTTTGTAAGCATGCTAAG
GAGAAGTCAGAAAGCGGAGACACGCTCAAATAGCAGTAACAACAATGGTTCTTCGTG
GTGGAGCTCCAGTCTTGTGAATGCAGCAACGAATTTCAAATCATCAGCCGAAGCTGG
AATGCAGAAGCTGAGCACCGTGACTAGCCAAGCCTTCCAGAAGGCTAAGGATGGGAT
CGCCCCAAATAGCAGCAGCAGCAGTGGTGACCTTGTGGAGCAATGTGTTCACTGCCC
AGCAAGATTTTCCACCGTGGGGGCCTTAATTGAACATGTTGAGAAATCCCACCAGATC
AACTCACAACCAAGTCATGGCCGAGTGACGATTGATGTTTGCCCAAAATGCAGCAAG
GCGTTCCGGGATCCTGTGTTGCTTGTGGAGCATGTTGAGAAGGAGCATGGAGGAACGT

CAAGAGTGTAG
>Zm00001d005698
ATGGCGCGGCGGGGCACGGAGGCGTTCCCTAACCTGGGCGCGCACTGCGACAAGCC
GGACTGCAACCAGCTCGACTTCCTGCCCTTCGACTGCGACGGCTGCGGCAAGGTCTTC
TGCGCCGCGCACCGGACCTACGGGGACCACGGCTGCGCCAAGGCGGCGGACCAGGG
CCGCACCGTCGTCGTCTGCCCGGACTGCGGCGACGCCATCGAGAGGCTGGTTCCGGG
GCAGGGCGAGCGGGAGATCCTGGAGGCGCACGTGCGGTCGCGGCGCTGCGACCCGG
GCAAGAAGCGCAAGCCCCGGTGCCCCGCGCGGCGGTGCAGGGAGCAGCTCACCTTC
TCCAACACCCAGGACTGCAAGGGCTGCGGCCGGAAGGTGTGCCTCAAGCACAGGTTC
CCGGCCGACCACGACTGCGCCGCCAGCGCACCAGGAGCTGCCGCGGCGGCGAGGAG
GGCCAGCGGGGAGTGCGGCCGCGGCGCGCGGAAGGAAGGGAGCGGCGGCTGGGCG
CTGCCGGCGTCCATCCGGAGCCTCAAGATCTTCTGA
>Zm00001d046767
ATGGAGCACAAGGAGGCTGGGTGCCAGGCCCCCGAGGGACCCATCCTCTGCATCAAT
AACTGTGGCTTCTTCGGCAGCGCGGCGACCATGAACATGTGCTCCAAGTGCCACAAG
GAGATGATAACGAAGCAGGATCAGGCCAAGCTGGCTGCCTCCTCTATCGACAGCATC
GTGAACGGCAGCGACGCCGTCATGGAGCCGGTTGTTGCTGGCAGCAACACGGTAGTA
GCTGTTGCCCAAGTTGAGTTGCAAACAATGAACGTGCAGCAGCCCGCTGATGTTGCC
GGACCCAGCGAGGGGGTGGCGGCGATCTCCAAAGGGGGGAAGGTAGGGCCGAACCG
GTGCAGCGCCTGCAGGAAGAGGGTTGGACTTACGGGATTCAACTGCCGGTGTGGGAA
CTTGTACTGCGCACTCCACCGCTACTCCGACAAGCACGACTGCAAGTTCGACTACCGG
ACTGCTGCCAGGGACGCCATTGCCAAGGCTAATCCGGTGGTGAAGGCAGACAAGCTC
GACAAGATCTAG
>Zm00001d006016
ATGGCGCAGCGCGACAAGAAGGAGGAGCCCACGGAGCTCCGGGCGCCGGAGATCAC
GCTGTGCGCCAACAACTGCGGGTTCCCGGGTAACCCGGCCACGCAGAACCTGTGCCA
GAGCTGCTTCTCGGCGTCGAGGTCGCCGTCGTCCCCTACCTCCTCCTCCTCTTCCCTGG
CGTCCGCGGCGTCGCAGCCGAGGCCAGCAGCGCTCGTCGTAGACGCGGCAGCGGTG
GAGGCGCTGGCCGCTCCGGCGGCCGCGGCCGTGGGCCAGGCTACGGAAGCCGCGGC
GAGGGCGTCCGCGAGCCGGTGCTCCAGCTGCCGGAAGCGCGTGGGGCTGACGGGGTT
CCGGTGCCGCTGCGGCGAGCTCTTCTGCGGCGCGCACCGGTACTCGGACCGGCACGG
CTGCAGCTACGACTACAGGGGCGCCGCCCGGGACGCCATCGCCCGGGAGAACCCGGT
GGTGCGCGCGGCCAAGGTCGTTAGGTTCTGA

>Zm00001d034389
MTQKRKSIGRGGVEDCGSPARAAACTSTTAASTSTTVEEKNTPAVFETTPPLWMTGPAETK
KPKIASSSSSSSSSSSSSPDGGSNNAAVAQAQPQPPPANRCSACRKKVGLLGFRCCCGKTFCG
AHRYAEKHACGFDYKHAGRGRIAKENPIIVADKIAKI
>Zm00001d053671
MNMCSKCHKEMIMKQEQAQLAASSIDSIVNGGDNGKGPAIAATVGVAVPQVEEKTIAVQP
MHVAETSEAAAVIAKAKEGPNRCATCRKRVGLTGFNCRCGNTYCSMHRYSDKHDCQFDY
RTAARDAIAKANPVVKAEKLDKI
>Zm00001d020926

MAQRDKKEEPTELRAPEIALCANNCGFPGNPATQNLCQSCFSASRSSSSSSQPSPTSSPSASA
PAAAVPQPRPALLDAALQLAPPAAAAGQPVEASARTSANRCSSCRKRVGLTGFRCRCGDL
FCGAHRYSDRHGCRYDYRGAARDAIARENPVVRAAKIVRF
>Zm00001d031423
MAQRDKKVEEPTELRAPELTLCANSCGFPGNPATNNLCQACFLAATASSSASASVSPPPPSS
SSSSPAVLQFDEQQQQQNPRPRAPAASGPTEEPPRPARASAPAPAPASSSSVRRCQTCRKRV
GLTGFRCRCGDLFCGAHRYSDRHDCCFDYRAAGRDAIARDNPVVRAAKIVRF
>Zm00001d015842
MEHKEAGCQQPEGPILCINNCGFFGSAATMNMCSKCHKEMITKQEQAQLAASSIDSIVNG
GDGGKGPVIAASVNVAVPQVEQKTIVVQPMLVAETSEAAAVIPKAKEGPDRCAACRKRVG
LTGFSCRCGNMYCSVHRYSDKHDCQFDYRTAARDAIAKANPVVRAEKLDKI
>Zm00001d021842
MGTPEFPNLGKHCSVGDCNQIDFLPFTCDRCDHVFCLDHRSYTSHQCPNANMKDVTVLIC
PLCAKGVRLNPSEDPNITWDTHVNTDCDPSNYQKVMKKKKCPVPGCRETLTFSNTIRCKD
CTREHCLKHRFGPDHKCPGPRKVDSGFPFVSMLRRSQKAETRSNSSNNNGSSWWSSSLVN
AATNFKSSAEAGMQKLSTVTSQAFQKAKDGIAPNSSSSSGDLVEQCVHCPARFSTVGALIE
HVEKSHQINSQPSHGRVTIDVCPKCSKAFRDPVLLVEHVEKEHGGTSRV
>Zm00001d005698
MARRGTEAFPNLGAHCDKPDCNQLDFLPFDCDGCGKVFCAAHRTYGDHGCAKAADQG
RTVVVCPDCGDAIERLVPGQGEREILEAHVRSRRCDPGKKRKPRCPARRCREQLTFSNTQD
CKGCGRKVCLKHRFPADHDCAASAPGAAAAARRASGECGRGARKEGSGGWALPASIRSL
KIF
>Zm00001d046767
MEHKEAGCQAPEGPILCINNCGFFGSAATMNMCSKCHKEMITKQDQAKLAASSIDSIVNG
SDAVMEPVVAGSNTVVAVAQVELQTMNVQQPADVAGPSEGVAAISKGGKVGPNRCSACR
KRVGLTGFNCRCGNLYCALHRYSDKHDCKFDYRTAARDAIAKANPVVKADKLDKI
>Zm00001d006016
MAQRDKKEEPTELRAPEITLCANNCGFPGNPATQNLCQSCFSASRSPSSPTSSSSSLASAASQ
PRPAALVVDAAAVEALAAPAAAAVGQATEAAARASASRCSSCRKRVGLTGFRCRCGELFC
GAHRYSDRHGCSYDYRGAARDAIARENPVVRAAKVVRF
