# Supplementary material for: Identification and Analysis of Stress-Associated Proteins (SAPs) Protein Family and Drought Tolerance of ZmSAP8 in Transgenic Arabidopsis
Source: Int J Mol Sci. 2022 Nov 15;23(22):14109. doi: 10.3390/ijms232214109 (PMC9696418; doi:10.3390/ijms232214109)
Supplement: Supplementary file 1 [file ijms-23-14109-s001.zip › supplymentary files.pdf]

Table S1 Genome information of plant species and outgroups

Taxonomy	Species name	Abrr.	Genome Size（Mb）	Gene Loci	Chromsome number	SAP number
Amborellaceae	Amborella trichopoda	Amtri	870	26846	13	7
Bromeliaceae	Ananas comosus	Ancom	440.8	29686	25	15
Brassicaceae	Arabidopsis thaliana	AT	119.7	27334	6	14
Poaceae	Brachypodium distachyon	Brdis	218.3	37892	5	14
Nematode	Caenorhabditis elegans	Caele	100	20512	6	4
Rutaceae	Citrus clementina	Cicle	301.365	32586	9	11
Cucurbitaceae	Cucumis sativus	Cusat	240.989	30919	7	13
Cyanidiaceae	cyanidioschyzon merolae	Cymer	16.5	5,373	20	1
Apiaceae	Daucus carota	Dacar	421.539	44655	9	9
Myrtoideae	Eucalyptus grandis	Eugra	653.98	46010	11	11
Fabaceae	Glycine max	Glmax	978	56044	20	27
Malvaceae	Gossypium raimondii	Gospp	761.252	55238	13	20
Panicoideae	Zea mays	Zemay	2191.6	57578	10	9
Rosaceae	Malus domestica	Madom	647.511	47440	17	32
Euphorbiaceae	Manihot esculenta	Maesc	708.18	49290	18	16
Marchantiaceae	Marchantia polymorpha	Mapol	222.246	24086	9	3
Trifolieae	Medicago truncatula	Metru	412.924	42683	8	17
Mamiellaceae	Micromonaspusilla CCMP1545	Mipus	22	10,248	19	1
Musaceae	Musa acuminata	Muacu	461.539	47707	11	22
Mammalia	Mus musculus	Muscu	2662.4	22619	21	11
Poaceae	Oryza sativa	Orsat	388.93	38007	12	18
Bryopsida	Physcomitrium patens	Phpat	472.081	48002	27	9
Salicaceae	Populus trichocarpa	potri	434.29	51717	19	19
Selaginellales	Selaginella moellendorffii	Semoe	212.315	45247	51	6
Poaceae	Setaria italica	Seita	441.705	35844	9	17
Solanaceae	Solanum tuberosum	Sotub	810.123	37999	12	17
Solanaceae	Solanum lycopersicum	Lyesc	805.374	37660	12	13
Andropogoneae	Sorghum Bicolor	SoBic	715.371	40148	10	16
Araceae	Spirodela polyrhiza	Sppol	137.856	19509	10	12
Malvaceae	Theobroma cacao	Thcac	335.437	37520	10	12
Hominidae	Homo sapiens	Hosap	3174.4	59,265	23	2

Table S2 Primer sequences used for experiments

Gene name	Forward primer(5′-3′)	Reverse primer(5′-3′)
qPCR-*Zm00001d020926*	TTCGCAGCCGTCCCCTAC	GTAGTCGTAGCGGCAGCC
qPCR-*Zm00001d031423*	AGTTCGACGAGCAGCAG	TGTAGTCGAAGCAGCAG
qPCR-*Zm00001d015842*	GGCGGGAAAGGACCTGTA	TAAATCCCGTCAGCCCAA
qPCR-*Zm00001d046767*	TGATAACGAAGCAGGATC	CTACCTTCCCCCCTTTGG
qPCR-*Zm00001d006016*	CAAGAAGGAGGAGCCCA	GCCAGGGAAGAGGAGGA
qPCR-*Zm00001d034389*	CGAAGAAGCCCAAGATC	TGATTGGGTTCTCCTTG
qPCR-*Zm00001d053671*	GTGATAATGGGAAAGGA	TCGGAGTAGCGGTGCAT
qPCR-*Zm00001d021842*	GACTTCCTCCCCTTCAC	TGTTGGAAAATGTCAGT
qPCR-*Zm00001d005698*	ACAAGCCGGACTGCAAC	TTGGAGAAGGTGAGCTG
1305-ZmSAP8	aagtccggagctagctctagaATGGGCACGCCGGAGTTC	ggtcctcgagacgtctctagaCTACACTCTTGACGTTCCTCCATG
1301-*ZmSAP8*	gagctcggtacccggggatccATGGGCACGCCGGAGTTC	caggtcgactctagaggatccCTACACTCTTGACGTTCCTCCATG
AD-*ZmSAP8*	gtgggcatcgatacgggatccATGGGCACGCCGGAGTTC	cagctcgagctcgatggatccCTACACTCTTGACGTTCCTCCATG
BD-*ZmSAP8*	aggccgaattcccggggatccATGGGCACGCCGGAGTTC	ccgctgcaggtcgacggatccCTACACTCTTGACGTTCCTCCATG

Table S3 Ka,Ks and Ka/Ks ration of WGD/SD and LD duplicated gene pairs

Gene 1	Gene 2	Ka	Ks	Ka/Ks
MDP0000874708	MDP0000133254	0.466757	6.135871	0.07607
MDP0000362677	MDP0000588934	0.676244	2.713958	0.249173
MDP0000516205	MDP0000707978	0.329273	1.144542	0.28769
MDP0000292844	MDP0000494946	0.479568	2.059687	0.232836
MDP0000211516	MDP0000263150	0.901293	3.239392	0.278229
MDP0000293524	MDP0000543745	0.914377	3.080917	0.296787
MDP0000294781	MDP0000165407	0.674836	3.311112	0.203809
MDP0000362676	MDP0000316313	0.176954	0.546418	0.323845
MDP0000294781	MDP0000165407	0.674836	3.311112	0.203809
MDP0000362676	MDP0000316313	0.176954	0.546418	0.323845
AT2G41835.1	AT3G57480.1	0.090349	1.129629	0.079981
AT2G36320.1	AT3G52800.1	0.137055	0.95467	0.143563
AT4G12040.1	AT4G22820.1	0.208224	0.908912	0.229091
AT2G41835.1	AT3G57480.1	0.090349	1.129629	0.079981
Bradi1g36050	Bradi3g07060	0.112359	0.892188	0.125937
Bradi1g06002	Bradi1g56250	0.340819	1.126378	0.30258
Bradi1g36050	Bradi3g07060	0.112359	0.892188	0.125937
Eucgr.A02408	Eucgr.K01813	0.307259	1.671868	0.183782
Eucgr.K01813	Eucgr.K01814	0.098875	0.524701	0.188442
Glyma.02G183100	Glyma.10G103400	0.018751	0.116061	0.161561
Glyma.03G137500	Glyma.10G103400	0.123334	0.869662	0.141818
Glyma.10G103400	Glyma.19G140400	0.134947	0.894457	0.15087
Glyma.03G194000	Glyma.10G070900	0.137067	0.903896	0.15164
Glyma.10G070900	Glyma.11G210000	0.057993	0.138177	0.419705
Glyma.10G070900	Glyma.19G193800	0.183942	0.730964	0.251644
Glyma.05G054400	Glyma.17G136800	0.047303	0.200073	0.236427
Glyma.09G156100	Glyma.16G206800	0.049733	0.153778	0.32341
Glyma.11G132000	Glyma.15G033400	0.092916	0.305724	0.303921
Glyma.12G056500	Glyma.15G033400	0.101307	0.320583	0.316009
Glyma.13G341000	Glyma.15G033400	0.046228	0.073341	0.630314
Glyma.13G204100	Glyma.15G272300	0.169762	1.00853	0.168326
Glyma.12G238400	Glyma.15G272300	0.171788	1.07934	0.159161
Glyma.13G069200	Glyma.19G013700	0.033774	0.214566	0.157407
Glyma.11G132000	Glyma.13G341000	0.081767	0.304639	0.268408
Glyma.08G153200	Glyma.13G204100	0.161453	1.040343	0.155192
Glyma.12G238400	Glyma.13G204100	0.037285	0.199887	0.186532
Glyma.02G183100	Glyma.03G137500	0.119245	0.859154	0.138793
Glyma.02G183100	Glyma.19G140400	0.123039	0.902347	0.136355
Glyma.03G194000	Glyma.19G193800	0.029665	0.132969	0.223096
Glyma.11G210000	Glyma.19G193800	0.176719	0.882372	0.200278
Glyma.12G183800	Glyma.19G193800	0.323986	1.375935	0.235466

Glyma.03G137500	Glyma.19G140400	0.03589	0.162957	0.220244
Glyma.03G140500	Glyma.19G143200	0.028202	0.104683	0.269409
Glyma.03G194000	Glyma.11G210000	0.160744	0.853024	0.188441
Glyma.03G194000	Glyma.12G183800	0.264884	1.816495	0.145821
Glyma.11G132000	Glyma.12G056500	0.01249	0.080195	0.155744
Glyma.08G153200	Glyma.12G238400	0.173068	0.873629	0.198102
Glyma.12G056500	Glyma.13G341000	0.090055	0.332821	0.270581
Gorai.002G240900.1	Gorai.009G404700.1	0.110483	0.682293	0.161928
Gorai.006G156200.1	Gorai.009G404700.1	0.085225	0.598861	0.142312
Gorai.001G121500.1	Gorai.009G086500.1	0.122783	0.703163	0.174616
Gorai.009G086500.1	Gorai.013G258400.1	0.170826	0.765711	0.223094
Gorai.004G036200.1	Gorai.004G173900.1	0.200315	1.275934	0.156995
Gorai.006G139100.1	Gorai.007G358100.1	0.101257	0.668098	0.15156
Gorai.002G229200.1	Gorai.007G358100.1	0.11548	0.842738	0.13703
Gorai.009G086500.1	Gorai.013G258400.1	0.170826	0.765711	0.223094
Gorai.001G121500.1	Gorai.013G258400.1	0.184518	0.687517	0.268383
Gorai.002G230400.1	Gorai.006G137400.1	0.102685	0.705948	0.145458
Gorai.002G229200.1	Gorai.007G358100.1	0.11548	0.842738	0.13703
Gorai.002G229200.1	Gorai.006G139100.1	0.130584	0.584061	0.223579
Gorai.002G240900.1	Gorai.009G404700.1	0.110483	0.682293	0.161928
Gorai.001G121500.1	Gorai.002G240900.1	0.323548	1.770161	0.182779
Gorai.002G240900.1	Gorai.006G156200.1	0.10625	0.750488	0.141575
Gorai.003G167800.1	Gorai.006G211300.1	0.077775	0.88059	0.088322
Gorai.003G167800.1	Gorai.008G240900.1	0.087591	0.760252	0.115213
Gorai.003G167800.1	Gorai.008G240900.1	0.087591	0.760252	0.115213
Gorai.006G211300.1	Gorai.008G240900.1	0.093723	0.677801	0.138276
Gorai.006G139100.1	Gorai.007G358100.1	0.101257	0.668098	0.15156
Medtr1g060380.1	Medtr2g086190.1	0.389378	1.552628	0.250786
Medtr1g060380.1	Medtr7g104320.1	0.250523	1.363301	0.183762
Medtr2g086190.1	Medtr4g053440.1	0.228965	1.59467	0.143582
Os08g39450	Os09g31200	0.198051	0.402396	0.492179
Os03g57890	Os07g07350	0.160083444	0.908485658	0.176209104
Os02g10200	Os06g41010	0.072584072	0.990505631	0.073279818
Os08g39450	Os09g31200	0.198051	0.402396	0.492179
Os03g57890	Os03g57900	0.437973	1.90539	0.22986
Pp3c7_310	Pp3c11_25720	0.152504	1.156748	0.131838
Pp3c5_22610	Pp3c6_6640	0.085016	0.885882	0.095967
Potri.001G269400.1	Potri.009G063900.1	0.080986	0.363638	0.222711
Potri.004G184300.1	Potri.009G144100.1	0.084819	0.504646	0.168075
Potri.001G115000.1	Potri.003G117100.1	0.074298	0.353143	0.210392
Potri.001G269300.1	Potri.003G205500.1	0.465226	1.710507	0.271982
Potri.001G018600.1	Potri.003G205500.1	0.043931	0.109701	0.400459
Potri.001G269300.1	Potri.006G056500.1	0.442871	2.215111	0.199932
Potri.006G056500.1	Potri.016G051700.1	0.028525	0.288242	0.098964

Potri.001G269300.1	Potri.016G051700.1	0.423424	1.642927	0.257725
Potri.001G115000.1	Potri.015G131900.1	0.537696	2.265847	0.237305
Potri.001G115000.1	Potri.012G130000.1	0.513735	1.82521	0.281466
Potri.001G018600.1	Potri.001G269300.1	0.484249	1.511779	0.320317
Potri.012G130000.1	Potri.012G130100.1	0.19418	0.391972	0.495391
Sobic.004G079100	Sobic.010G190600	0.109325	1.114612	0.098084
Sobic.002G245800	Sobic.007G212200	0.199405	0.414411	0.481178
Sobic.001G062200	Sobic.002G046100	0.480652	1.127276	0.426384
Sobic.007G138100	Sobic.007G138200	0.163365	0.192982	0.84653
Sobic.007G138000	Sobic.007G138100	0.063872	0.129957	0.491484
Sobic.002G046000	Sobic.002G046100	0.054189	0.352115	0.153895
Seita.2G252300.1	Seita.6G203700.1	0.198519	0.46969	0.422661
Seita.2G045100.1	Seita.9G061100.1	0.414121615	1.209838327	0.342295004
Seita.5G232000.1	Seita.5G232100.1	0.083457944	0.216074511	0.386246132
Spipo10G0023600	Spipo12G0034400	0.493836	1.400572	0.352596
Spipo0G0006000	Spipo13G0001900	0.228901	0.742939	0.308102
Spipo0G0006000	Spipo9G0034900	0.225684	0.848755	0.2659
Spipo7G0052000	Spipo9G0054100	0.225773	0.814465	0.277204
Solyc09g009590.1.1	Solyc10g083460.1.1	0.283194	1.780128	0.159086
Solyc01g086970.2.1	Solyc10g079080.1.1	0.252224	2.434044	0.103624
Solyc04g015570.2.1	Solyc10g080200.1.1	0.149586	0.693712	0.215632
Zm00001d031423	Zm00001d020926	0.163976	0.576868	0.284253
Zm00001d053671	Zm00001d015842	0.05375	0.265963	0.202094
Zm00001d031423	Zm00001d006016	0.213516	0.50648	0.421568
Zm00001d006016	Zm00001d020926	0.100218	0.262735	0.38144

Table S4 Length of RT-qPCR product

Gene ID v4	length of PCR product
Zm00001d020926	270 bp
Zm00001d031423	250 bp
Zm00001d015842	187 bp
Zm00001d046767	213 bp
Zm00001d006016	159 bp
Zm00001d034389	262 bp
Zm00001d053671	218 bp
Zm00001d021842	283 bp
Zm00001d005698	289 bp
